# SSR-Linkage map of interspecific populations derived from *Gossypium trilobum* and *Gossypium thurberi* and determination of genes harbored within the segregating distortion regions

**DOI:** 10.1371/journal.pone.0207271

**Published:** 2018-11-12

**Authors:** Pengcheng Li, Joy Nyangasi Kirungu, Hejun Lu, Richard Odongo Magwanga, Pu Lu, Xiaoyan Cai, Zhongli Zhou, Xingxing Wang, Yuqing Hou, Yuhong Wang, Yanchao Xu, Renhai Peng, Yingfan Cai, Yun Zhou, Kunbo Wang, Fang Liu

**Affiliations:** 1 State Key Laboratory of Cotton Biology /Institute of Cotton Research, Chinese Academy of Agricultural Science, Anyang, Henan, China; 2 School of Life Science, Henan University/State Key Laboratory of Cotton Biology/Henan Key Laboratory of Plant Stress Biology, Kaifeng, Henan, China; 3 School of Biological and Physical Sciences (SBPS), Jaramogi Oginga Odinga University of Science and Technology (JOOUST), Bondo- Kenya; 4 Biological and Food Engineering, Anyang Institute of technology, Anyang, Henan, China; USDA-ARS Southern Regional Research Center, UNITED STATES

## Abstract

Wild cotton species have significant agronomic traits that can be introgressed into elite cultivated varieties. The use of a genetic map is important in exploring, identification and mining genes which carry significant traits. In this study, 188 F_2_mapping individuals were developed from *Gossypium thurberi* (female) and *Gossypium trilobum* (male), and were genotyped by using simple sequence repeat (SSR) markers. A total of 12,560 simple sequence repeat (SSR) markers, developed by Southwest University, thus coded SWU were screened out of which only 994 were found to be polymorphic, and 849 markers were linked in all the 13 chromosomes. The map had a length of 1,012.458 cM with an average marker distance of 1.193 cM. Segregation distortion regions (SDRs) were observed on Chr01, Chr02, Chr06, Chr07 Chr09, Chr10 and Chr11 with a large proportion of the SDR regions segregating towards the heterozygous allele. There was good syntenic block formation that revealed good collinearity between the genetic and physical map of *G*. *raimondii*, compared to the Dt_sub genome of the *G*. *hirsutum* and *G*. *barbadense*. A total of 2,496 genes were mined within the SSR related regions. The proteins encoding the mined genes within the SDR had varied physiochemical properties; their molecular weights ranged from 6.586 to 252.737 kDa, charge range of -39.5 to 52, grand hydropathy value (GRAVY) of -1.177 to 0.936 and isoelectric (pI) value of 4.087 to 12.206. The low GRAVY values detected showed that the proteins encoding these genes were hydrophilic in nature, a property common among the stress responsive genes. The RNA sequence analysis revealed more of the genes were highly upregulated in various stages of fiber development for instance; *Gorai*.*002G241300* was highly up regulated at 5, 10, 20 and 25 day post anthesis (DPA). Validation through RT-qPCR further revealed that these genes mined within the SDR regions might be playing a significant role under fiber development stages, therefore we infer that Gorai.007G347600 (*TFCA*), Gorai.012G141600 (*FOLB1*), Gorai.006G024500 (*NMD3*), Gorai.002G229900 (*LST8*) and Gorai.002G235200 (*NSA2*) are significantly important in fiber development and in turn the quality, and further researches needed to be done to elucidate their exact roles in the fiber development process. The construction of the genetic map between the two wild species paves away for the mapping of quantitative trait loci (QTLs) since the average distance between the markers is small, and mining of genes on the SSR regions will provide an insight in identifying key genes that can be introgressed into the cultivated cotton cultivars.

## Introduction

Cotton (*Gossypium* spp.) is the most important fiber crop and one of the sources for animal feeds and edible oil. However cotton growing has significantly been threatened by various abiotic and biotic stresses, this condition has been worsened by intensive selection and inbreeding resulting into the narrow genetic base [1]. Improving cotton yield and fiber quality is important for the survival of the cotton industry [2]. Serious outbreaks of diseases and pests have resulted in great loss in fibre production and its quality.

Researchers are facing difficulties in developing new varieties of cotton to meet emerging challenges, this is because of the limited diversity in the Germplasm of the commercially cultivated, upland cotton [3]. Wild cotton species have rich reservoir of genetic material, much of which has potential and valuable agronomic traits [4]. Some of the useful alleles introgressed into elite cultivars have been achieved through interspecific hybridization for instance, improvement in fibre quality where, long fibre length upland cotton, *G*. *hirsutum* was achieved through tri-interspecific hybridization between *G*. *thurberi*, *G*. *raimondii* and *G*. *barbadense* [5]. Drought resistance in upland cotton has been achieved through the utilization of alleles from Asiatic Cottons [6]. Production of fertile hybrid germplasm with diploid Australian *Gossypium* species has been achieved [7].

Cotton are of two types, the diploid and the tetraploid species, the diploid have 13 chromosomes, n = 2n = 26, while the tetraploid cotton emerged due to whole genome duplication of the two diploid parental lines, resulting into 2n = 4n = 52 chromosomes [8]. The diploid cotton are subdivided into A,B, C, D,E,F,G and K genomes, on the other hand, there are 7 known species of AD genome [9]. Among the diploid cotton genomes, D genome has been found to harbor high number of significant agronomic traits, such as superior fiber qualities, and high tolerance to both biotic and abiotic stresses [10,11]. In a number studies done on the tetraploid cotton, more on quantitative trait loci (QTL) mapping, high number of QTLSs have been found to be mapped on the Dt_sub genome compared to At_sub genome [12–15]. This explains the significance on the D genome and ability to utilize the genes in D cotton species to improve the elite cotton cultivars, which have been has narrow genetic base due to intensive selection and inbreeding [16–18]. The two wild cotton species, *G*. *thurberi* and *G*. *trilobum* belong to the diploid cotton of the D genome. *G*. *thurberi* Todaro (D_8_) is a wild cotton species, native to Mexico in the Sonora Desert and parts of the southwestern part of the United States of America (USA). *G*. *thurberi* has good characteristics that can be introgressed into elite cultivars such as fibre fineness, fibre strength, long fibre, prolific boll bearing, resistance to *Fusarium wilt*, resistance to frost and cotton bollworms [19]. In addition, *G*. *thurberi* has been found to be highly resistant to silver leaf whitefly [20]. The second parental line used in this research, *G*. *trilobum* (D_1_) is an endemic species of West and central Mexico. It has glabrous leaves which is a key character for its identification, it has important agronomic traits such as resistance to *Verticillium wilt* and drought tolerance [21].

The application of genetic maps between interspecific crosses in cotton, have become vital tools in understanding the genome structure, exploring important agronomic traits and also provide the basis for finding new DNA markers for further construction of high density maps [22]. Currently there are limited numbers of genetic maps that have been constructed from interspecific crosses between the wild progenies of the D cotton genome. The use of Simple sequence repeats (SSRs) are considered to be one of the markers of choice for genome mapping, because they are PCR-based, co-dominancy, multiallelic and hyper-variable in nature [23]. SSR markers, derived from either genomic region or expressed sequence tags (EST), are considered to be essential in the construction of genetic maps. In addition, EST-SSR markers have been extensively used in unraveling the complexities of eukaryotic organisms genomes being that they are directly tagged to the functional genes [24]

In this study we developed an F_2_generation between two wild cotton species in the D genome; *G*. *thurberi* and *G*. *trilobum*. We applied the use of mono-markers, SWU simple sequence repeat (SSR) in genotyping 188 individuals of the F_2_generation. The developed genotypes were applied in the construction of the genetic map; the map enabled us to unearth some of the vital transcriptome factors with profound effect on fiber development. The linkage map and the genes mined will provide a basis in genetic studies such as Marker-assisted selection (MAS) and gene transformation.

## Materials and methods

### Parental materials

*G*. *thurberi* as female parent was crossed with *G*. *trilobum* as male parent to obtain F_1_ generation. The F_1_ generation was then self-pollinated to get the F_2_individuals. A total of 274 F_2_were obtained through F_1_ self-crossing. From the F_2_progenies, 188 individuals were randomly selected for genotypic analysis with the polymorphic markers. The two parental materials and the F_2_ progenies were developed at the National Wild Cotton Nursery in Sanya, Hainan Island, China.

### DNA extraction, quantification and electrophoresis

The leaves from the parents, F_1_ individual and F_2_progenies were collected and stored in the fridge at -80°C. DNA extraction was done following the CTAB method [25]. DNA quantification and purification was then done to determine the concentration and level of RNA contamination using the Nanodrop techniques, Spectrophotometer was used for quantification and quality checking depending on A260/A280 [26]. Concentration of genomic DNA was estimated by comparing the size and intensity of each sample band with those of sizing standard, DNA mass ladder. We then diluted the sample according to each sample concentration until it was within the working concentration range; the DNA working concentration was based on 10–100 μg/μl. The polymerase chain reaction (PCR) amplification on the reagents was conducted using TAKARA Bio Inc TP 600 thermal cycler. Electrophoresis was performed on the PCR product following the method described by [25] with minor modifications. The amplified PCR products were separated on 8% denaturing polyacrylamide gel and visualized by silver nitrate staining [27].

### Application of SSR markers genotyping the F_2_progenies derived from the two diploid parental lines

We employed the use of expressed sequence tag-simple sequence repeat (EST-SSR) mono-markers developed by South West University, China thus the acronym SWU. The SWU markers were developed from *G*. *raimondii* genome. A total of 12,650 markers were screened for polymorphism, out of this we obtained 996 polymorphic loci which were used to genotype 188 F_2_ individuals, the Details of the SWU markers, forward and reverse sequence are summarized in (S1 Table). The male plant *G*. *trilobum*, the female plant *G*. *thurberi* and the heterozygous F_2_ progenies were scored as A, B, and H respectively, The missing data was designated as‘-’.Multi-allelic markers were named separately by primer name followed by the letters a, b, c, and d as a suffix.

### Genetic map construction

We employed the use of Join Map 4.0 with a recombination frequency of 0.40 and a LOD score of 2.5 for the formation of linkage groups [28]. Linkage groups were assigned to chromosomes depending on blast searches on the markers since these markers are newly developed. The linkage groups were then drawn using Mapchart 2.3 Software [29], A Chi-square (χ2) test was performed to determine whether the markers significantly deviated from Mendelian segregation ratios The markers showing segregation distortion were indicated by asterisks (*P<0.05, **P<0.01, ***P<0.00, ****P<0.001, *****P<0.0005, ******P<0.0001, *******P<0.00005. The markers that deviated significantly from the normal Mendelian ratio of 3:1 for dominant markers and 1:2:1 for codominant markers were termed to be segregated distorted markers and were used to determine segregation distortion in the linkage groups [30].

### Gene mining, protein characterization and GO functional annotation

The physical positions of the flanking markers were employed in mining the genes. The SSR marker sequences were used as the query by blasting in to the reference genome, *G*. *raimondii* genome assembly, being the markers were developed from *G*. *raimondii*. By employing the physical position and use of cotton genome database, all the genes were obtained per each chromosome. The method adopted was similar to previous method employed by Magwanga et al [11] in obtaining the conserved genes between two tetraploid cotton, *G*, *hirsutum* and *G*. *tomentosum*. Further analysis were carried out on the various genes mined in order to determine the characteristics of the proteins encoding the mined genes and their putative roles in cotton through GO annotation, which was carried out through BLAST2GO [31]. Furthermore, the isoelectric points (pI), grand hydropathy values (GRAVY), charge and molecular masses of the proteins encoding the mined genes were estimated by ExPASy Server tool (http://web.expasy.org/compute_pi/).

### RNA expression analysis and RT-qPCR validation of the highly upregulated genes

We obtained the RNA sequence data for the genes mined within the SDR regions. The RNA-seq data was obtained from the Cotton Functional Genomics Database (https://cottonfgd.org/). The RNA sequenced data were for the reference genome, *G*. *raimondii* profiled at different stages of fiber development. The Raw RNA seq data were transformed into log 2, and used in the construction of heatmap. Furthermore, we selected 50 highly upregulated genes, and carried out RT-qPCR analysis in order to validate the possible role of these mined genes in fiber development using their gene specific primers (S2 Table). The two parental lines flowers were tagged and samples harvested at 0, 5, 25 and 30 DPA for real time quantitative polymerase chain reaction (RT-qPCR). The RT-qPCR analysis was carried out as outlined by Magwanga et al [1], cotton *GrActin* with forward sequence “ATCCTCCGTCTAGACCTTG” and reverse sequence “TGTCCATCAGGCAACTCAT” was used as the reference gene

### Collinearity analysis

A BLASTN Search with E ≤ 1 × 10^−5^, identity ≥ 80%, and matched length ≥ 200 bp was applied (https://blast.ncbi.nlm.nih.gov/Blast.cgi). The SSR sequences were used as queries, the genome assemblies of (AD)1 [32] genome and (AD)2 [33] were used in collinearity analysis. Markers with the best hits were chosen and the Circos program (http://circos.ca) was applied to draw the circos maps.

## Results

### Parental polymorphism

The SWU primers used were 12,560 in total and were used for screening for interspecific polymorphism between the two parental lines, *G*. *trilobum*, *G*. *thurberi* and their F_1_ generation. A total of 994 markers were obtained as polymorphic which accounted for only 7.91% of all the markers screened. A total of 132 (13.3%) markers were scored as dominant markers while 862 (86.7%) markers were scored as codominant. In our study we noted that the rate of polymorphism in the eSSRs markers used was lower this could be due to DNA sequences conserved at transcribed regions [34]. Low levels of polymorphism have been reported in other plants for instance in peanut (6.8%) polymorphism was detected among the eSSRs, [35], maize (1.4%), rice (4.7%), sorghum (3.6%), wheat (3.2%) [36], in *Gossypium* species lower polymorphic rate of the eSSRs have been reported [37–39]. However the eSSRs remain to be the markers of choice due to their ability to detect incomplete dominance inheritance, cost less and having a good genomic coverage despite the lower polymorphism observed in some plant [40].

### Genetic map construction and determination of the segregation distortion regions (SDRs)

**Fig 1:**
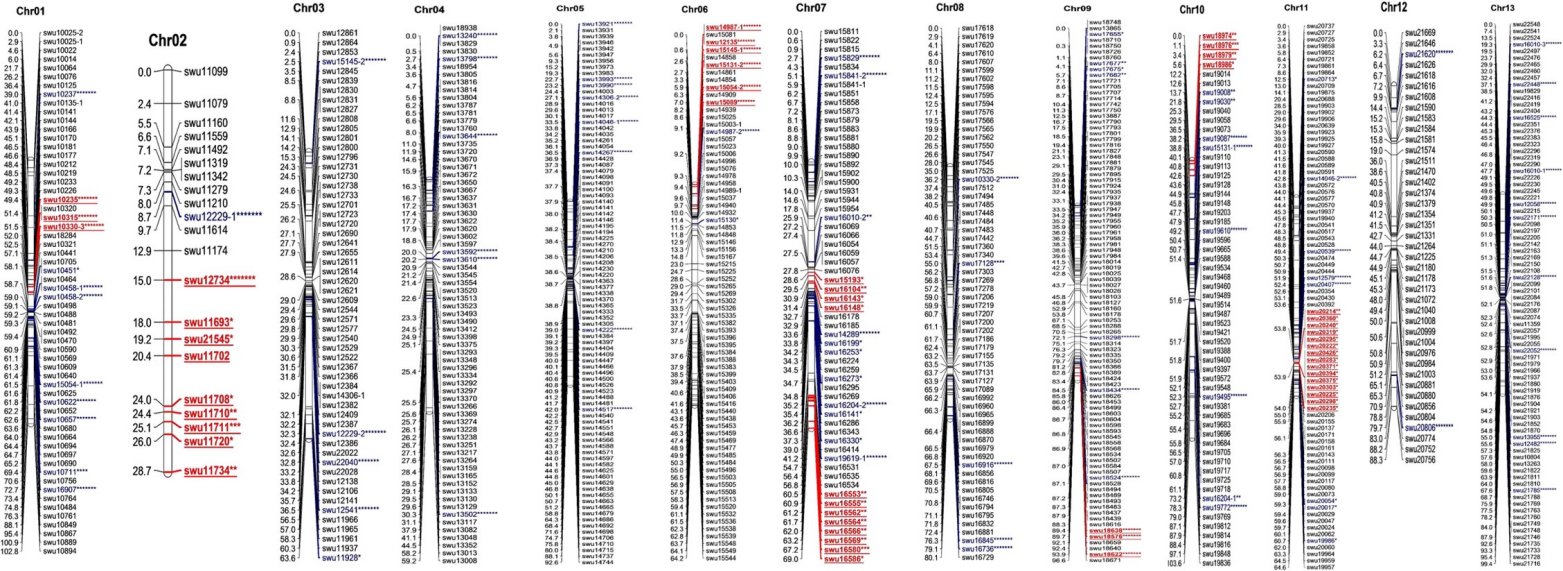
Genetic maps for the 13 chromosomes of the F_2_interspecific individuals derived between *G*. *thurberi* and *G*. *trilobum*. The markers in blue are distorted while markers in red and underlined indicates the distorted regions per chromosomes.

All the polymorphic markers used in the genotyping of the F_2_progenies were successfully scored and utilized in the construction of the linkage map by the use of JoinMap. A total of 849 out 994 polymorphic markers were linked and distributed across the entire 13 chromosomes of the D genome (Fig 1.). The details for the SSR markers and the alleles scores used for the construction of the genetic map are shown in (S3 Table). The distribution of markers on the linkages was symmetric and there was no clustering of loci, 145 loci were not linked due to high distortions. The genetic map size generated was 1,012.458 cM with an average marker distance of 1.193 cM. The chromosome with the highest marker loci density was Chr09 with 93 (11%) markers followed by Chr05 with 89 (10.5%) markers while Chr02 had the least number of markers loci with only 21 markers. The largest gap between adjacent loci was observed on Chr01 covering 15.699 cM while Chr04, Chr08, Chr10 and Chr11 had the smallest gap of 0.001 cM (Table 1). The longest chromosome was Chr12 spanning a distance of 103.563 cM while the shortest chromosome was Chr02 with a map distance of 28.665cM. A total of 714 loci (84.216%) accorded with the Mendelian ratio while 135 (15.783%) deviated from Mendelian ratio, chromosomes with the highest number of distorted loci were Chr07 and Chr11 with 23 distorted loci each while Chr12 had the least number of distorted loci with only 2 distorted loci (Table 1). Some regions on linkage groups had large clustered segregation distortion loci (SDLs); these regions were referred as segregation regions (SDR’S). A total of 8 SDR regions were noted on Chr01, Chr02, Chr06, Chr09, Chr10 and Chr11 each had a single SDR, designated as SDR1, SDR2, SDR6, SDR9, SDR10, and SDR11 respectively while Chr07 had two SDR’S namely SDR7-1, SDR7-2. Large clusters of segregated distorted loci on these regions were observed on Chr02, Chr06, Chr07 and Chr11. The largest SDR’s were skewed towards the heterozygous allele (Table 2).

**Table 1:** Characteristics of the genetic map.

Chro.	Mapped markers	Map Size (cM)	Av. Map distance (cM)	Gaps (cM) per Chromosome			Segregation Distortion				
				Smallest gap (cM)	Largest Gap (cM)	<10 cM	Ave. %SDs	*G*. *thurberi*	*G*. *trilobum*	Toward heterozygote	Number of SD
Chr01	60	102.761	1.713	0.018	15.699	54	20	5	4	3	12
Chr02	21	28.665	1.365	0.003	3.554	20	42.857	2	1	7	10
Chr03	56	63.601	1.136	0.004	19.969	52	8.929	0	4	1	5
Chr04	70	59.229	0.846	0.001	13.685	59	8.571	1	5	0	6
Chr05	89	92.563	1.04	0.002	8.12	80	8.989	2	5	1	8
Chr06	73	64.213	0.88	0.002	8.255	69	10.959	4	3	1	8
Chr07	60	69.003	1.15	0.006	13.503	57	38.333	4	1	18	23
Chr08	64	80.053	1.251	0.001	7.053	60	7.813	2	1	2	5
Chr09	93	96.559	1.038	0.004	13.289	81	10.753	4	2	4	10
Chr10	58	103.563	1.786	0.001	12.157	46	20.69	3	2	7	12
Chr11	82	64.604	0.788	0.001	8.104	71	28.049	4	0	19	23
Chr12	41	88.288	2.153	0.067	16.95	34	4.878	1	1	0	2
Chr13	82	99.356	1.212	0.002	7.421	65	13.415	6	5	0	11
Totals	**849**	**1012.46**	**1.193**	**0.009**	**11.366**	**748**	**15.783**	**38**	**34**	**63**	**135**

SD: segregation distortion; cM: centiMorgans; G: *Gossypium*; Chr: chromosome

**Table 2:** Analysis of the protein structure and domain features of the genes located within the SDRs.

Gene ID	Domain	Gene Name	Description	Chro.	Start	End	Len. (bp)	Exon No.	Mean Exon Len. (bp)	Mean Intron Len. (bp)
Gorai.002G229500	-	*-*	-	Chr02	58,870,423	58,870,953	531	1	531	intronless
Gorai.002G229600	-	*AGP23*	Arabinogalactan peptide 23	Chr02	58,874,911	58,875,444	534	1	534	intronless
Gorai.002G229700	-	*-*	-	Chr02	58,886,296	58,886,637	342	1	342	intronless
Gorai.002G235600	-	*-*	-	Chr02	59,612,406	59,614,356	1,951	4	329.3	211.3
Gorai.002G235700	-	*-*	-	Chr02	59,615,961	59,616,590	630	1	630	intronless
Gorai.006G069500	-	*-*	-	Chr06	28,104,135	28,105,057	923	2	394.5	134
Gorai.006G099600	-	*AGD14*	Probable ADP-ribosylation factor GTPase-activating protein AGD14	Chr06	34,010,085	34,014,482	4,398	8	208.1	390.4
Gorai.007G221700	-	*-*	-	Chr07	25,764,600	25,767,676	3,077	3	691.3	501.5
Gorai.007G347400	-	*-*	-	Chr07	57,766,119	57,766,961	843	2	304.5	234
Gorai.007G348800	-	*-*	-	Chr07	57,896,009	57,902,120	6,112	13	185.9	307.9
Gorai.007G349100	-	*CLE-4A-1*	CLAVATA3/ESR (CLE)-related protein 4A-1	Chr07	57,924,593	57,926,084	1,492	1	1,492.00	intronless
Gorai.007G349300	-	*-*	-	Chr07	57,944,764	57,948,953	4,190	10	113.4	339.6
Gorai.007G355900	-	*-*	-	Chr07	58,647,174	58,648,236	1,063	2	458.5	146
Gorai.009G367400	-	*-*	-	Chr09	49,208,616	49,209,005	390	1	390	intronless
Gorai.009G374800	-	*rbm48*	RNA binding protein 48	Chr09	50,946,132	50,950,973	4,842	7	126.3	374.8
Gorai.010G009800	-	*-*	-	Chr10	718,711	720,437	1,727	2	318	1,091.00
Gorai.011G137000	-	*SS4*	Probable starch synthase 4, chloroplastic/amyloplastic	Chr11	21,004,037	21,007,418	3,382	4	93.8	729
Gorai.011G137600	-	*-*	-	Chr11	21,157,120	21,157,891	772	1	772	intronless
Gorai.011G137700	-	*-*	-	Chr11	21,161,530	21,162,074	545	1	545	No intron
Gorai.011G137900	-	*-*	-	Chr11	21,170,291	21,172,601	2,311	5	237.8	280.5
Gorai.011G141300	-	*-*	23 kDa jasmonate-induced protein	Chr11	22,263,233	22,264,410	1,178	2	539.5	99
Gorai.011G142900	-	*-*	-	Chr11	22,543,659	22,549,407	5,749	7	209.1	374.2
Gorai.012G141700	-	*-*	-	Chr12	31,509,025	31,511,304	2,280	4	301.3	358.3
Gorai.006G021800	PF00010	*PRE5*	Transcription factor PRE5	Chr06	5,591,652	5,593,108	1,457	3	178.3	416.5
Gorai.007G349000	PF00025	*-*	ADP-ribosylation factor	Chr07	57,911,136	57,917,575	6,440	7	193.6	829.8
Gorai.007G346300	PF00046	*WOX11*	WUSCHEL-related homeobox 11	Chr07	57,419,364	57,421,615	2,252	3	393.3	536
Gorai.001G088900	PF00067	*CYP81D1*	Cytochrome P450 81D1	Chr01	9,602,033	9,604,342	2,310	2	874	562
Gorai.001G089000	PF00067	*CYP81E8*	Cytochrome P450 81E8	Chr01	9,618,655	9,620,814	2,160	7	149.1	186
Gorai.002G241100	PF00067	*CYP94C1*	Cytochrome P450 94C1	Chr02	60,454,083	60,455,817	1,735	1	1,735.00	intronless
Gorai.006G081800	PF00067	*-*	Cytochrome P450 CYP736A12	Chr06	31,044,712	31,046,253	1,542	3	439	112.5
Gorai.007G347700	PF00067	*CYP89A2*	Cytochrome P450 89A2	Chr07	57,779,914	57,781,455	1,542	1	1,542.00	intronless
Gorai.007G347800	PF00067	*CYP89A2*	Cytochrome P450 89A2	Chr07	57,788,882	57,790,696	1,815	1	1,815.00	intronless
Gorai.011G158300	PF00069	*LECRKS2*	Receptor like protein kinase S.2	Chr11	28,179,900	28,182,398	2,499	1	2,499.00	intronless
Gorai.011G162200	PF00069	*BAK1*	BRASSINOSTEROID INSENSITIVE 1-associated receptor kinase 1	Chr11	30,386,854	30,389,231	2,378	4	281	418
Gorai.009G374600	PF00071	*RABA1F*	Rasnarelated protein RABA1f	Chr09	50,937,237	50,940,126	2,890	2	543	1,804.00
Gorai.006G021900	PF00076	*BPA1*	Binding partner of ACD11 1	Chr06	5,598,552	5,602,965	4,414	5	373.4	556.3
Gorai.011G142700	PF00076	*-*	-	Chr11	22,525,363	22,528,458	3,096	3	829	304.5
Gorai.010G009900	PF00083	*At1g75220*	Sugar transporter ERD6-like 6	Chr10	725,683	731,830	6,148	19	109.6	223.8
Gorai.011G154700	PF00083	*PHT1-5*	Probable inorganic phosphate transporter 1–5	Chr11	26,839,390	26,841,160	1,771	1	1,771.00	intronless
Gorai.007G347300	PF00140	*SIGB*	R- polymerase sigma factor sigB	Chr07	57,758,649	57,762,734	4,086	8	273.8	270.9
Gorai.012G141500	PF00141	*poxN1*	Peroxidase N1	Chr12	31,500,409	31,501,774	1,366	3	400.7	82
Gorai.007G350800	PF00179	*UBC22*	Ubiquitin-conjugating enzyme E2 22	Chr07	58,098,369	58,102,739	4,371	7	170	502.3
Gorai.011G137500	PF00182	*EP3*	Endochitinase EP3	Chr11	21,155,488	21,156,581	1,094	2	502.5	89
Gorai.012G141400	PF00223	*psaA*	Photosystem I P700 chlorophyll a apoprotein A1	Chr12	31,499,254	31,499,712	459	2	219	21
Gorai.007G353100	PF00225	*CENPE*	Centromere-associated protein E	Chr07	58,374,076	58,394,945	20,870	34	135.8	492.5
Gorai.007G346400	PF00249	*MYB44*	Transcription factor MYB44	Chr07	57,434,322	57,436,192	1,871	3	565	88
Gorai.007G348600	PF00249	*MYB39*	Transcription factor MYB39	Chr07	57,887,881	57,889,534	1,654	4	302.8	147.7
Gorai.010G010000	PF00249	*RL6*	Protein RADIALIS-like 6	Chr10	734,790	736,367	1,578	2	144	1,290.00
Gorai.002G231300	PF00282	*SDC*	Serine decarboxylase	Chr02	59,077,239	59,080,226	2,988	5	426.2	195.5
Gorai.002G235500	PF00293	*NUDT27*	Nudix hydrolase 27, chloroplastic	Chr02	59,603,474	59,606,648	3,175	6	299.2	276
Gorai.006G024400	PF00295	*At1g80170*	Probable polygalacturonase At1g80170	Chr06	6,292,096	6,294,261	2,166	8	151.1	136.7
Gorai.006G023400	PF00328	*PPIP5K1*	Inositol hexakisphosphate and diphosphoinositol-pentakisphosphate kinase 1	Chr06	6,109,671	6,122,787	13,117	29	135.8	325.8
Gorai.001G122300	PF00332	*At2g27500*	Glucan endo-1,3-beta-glucosidase 14	Chr01	15,285,802	15,288,965	3,164	4	455	111
Gorai.007G347200	PF00385	*LHP1*	Chromo domain-containing protein LHP1	Chr07	57,754,602	57,758,058	3,457	6	357.8	262
Gorai.002G229900	PF00400	*lst8*	Protein LST8 homolog	Chr02	58,908,149	58,913,518	5,370	11	151.9	369.9
Gorai.003G137300	PF00403	*ATX1*	Copper transport protein ATX1	Chr03	39,650,473	39,651,557	1,085	3	302.7	88.5
Gorai.002G241200	PF00428	*RPP2B*	60S acidic ribosomal protein P2B	Chr02	60,460,978	60,462,786	1,809	4	190.3	349.3
Gorai.002G235100	PF00534	*DGD1*	Digalactosyldiacylglycerol synthase 1, chloroplastic	Chr02	59,555,124	59,562,274	7,151	7	429	691.3
Gorai.010G007600	PF00590	*rsmI*	Ribosomal R- small subunit methyltransferase I	Chr10	482,439	486,710	4,272	11	158.5	252.9
Gorai.002G229800	PF00595	*CTPA3*	Carboxyl-terminal-processing peptidase 3, chloroplastic	Chr02	58,890,304	58,897,730	7,427	12	185	473.4
Gorai.011G135300	PF00612	*IQD14*	Protein IQ-DOMAIN 14	Chr11	20,432,093	20,437,539	5,447	7	314	541.5
Gorai.009G367200	PF00646	*TULP7*	Tubby-like F-box protein 7	Chr09	49,203,689	49,207,350	3,662	4	441.8	631.7
Gorai.007G221600	PF00651	*At5g67385*	BTB/POZ domain-containing protein At5g67385	Chr07	25,760,938	25,764,226	3,289	5	539.8	147.5
Gorai.011G168600	PF00654	*CLC-D*	Chloride channel protein CLC-d	Chr11	34,350,554	34,362,793	12,240	23	164.3	382.7
Gorai.001G121600	PF00777	*GALT29A*	Beta-1,6-galactosyltransferase GALT29A	Chr01	14,837,936	14,840,197	2,262	1	1,191.00	intronless
Gorai.003G137400	PF00831	*RPL35*	60S ribosomal protein L35	Chr03	39,654,703	39,656,209	1,507	4	196.5	240.3
Gorai.007G356000	PF00931	*At4g27220*	Probable disease resistance protein At4g27220	Chr07	58,647,540	58,664,752	17,213	8	931.6	557
Gorai.006G069600	PF01015	*-*	40S ribosomal protein S3a	Chr06	28,109,329	28,112,694	3,366	7	177.3	354.2
Gorai.007G347100	PF01113	*DAPB2*	4-hydroxy-tetrahydrodipicoli-te reductase 2, chloroplastic	Chr07	57,748,162	57,753,918	5,757	9	132.9	570.1
Gorai.001G121800	PF01161	*CET2*	CEN-like protein 2	Chr01	15,030,129	15,031,205	1,077	4	145	165.7
Gorai.011G137100	PF01169	*At1g68650*	GDT1-like protein 5	Chr11	21,017,320	21,017,693	374	2	96	182
Gorai.002G235200	PF01201	*nsa2*	Ribosome biogenesis protein NSA2 homolog	Chr02	59,562,732	59,565,531	2,800	10	132.2	162.2
Gorai.011G181800	PF01326	*PPD*	Pyruvate, phosphate dikinase, chloroplastic	Chr11	43,199,802	43,207,641	7,840	21	161.6	222.3
Gorai.011G136900	PF01336	*At3g11710*	Lysine-tRNA ligase, cytoplasmic	Chr11	20,997,065	21,003,264	6,200	17	138.8	232.4
Gorai.002G231600	PF01370	*TKPR2*	Tetraketide alpha-pyrone reductase 2	Chr02	59,112,375	59,118,713	6,339	6	223.7	999.4
Gorai.007G349200	PF01373	*BMY1*	Beta-amylase	Chr07	57,929,522	57,934,365	4,844	8	237.5	420.6
Gorai.002G234600	PF01397	*CAD1-A*	(+)-delta-cadinene synthase isozyme A	Chr02	59,497,295	59,509,754	12,460	6	326.2	2,099.20
Gorai.006G099700	PF01412	*AGD14*	Probable ADP-ribosylation factor GTPase-activating protein AGD14	Chr06	34,014,603	34,015,470	868	3	100	284
Gorai.010G007400	PF01419	*JAL3*	Jacalin-related lectin 3	Chr10	450,000	455,270	5,271	7	277.9	554.3
Gorai.009G366600	PF01436	*-*	-	Chr09	49,122,684	49,126,097	3,414	7	281.3	240.8
Gorai.010G012200	PF01457	*GA17800*	Leishmanolysin-like peptidase	Chr10	943,286	949,671	6,386	17	202.1	184.4
Gorai.006G024700	PF01459	*-*	Mitochondrial outer membrane protein porin of 34 kDa	Chr06	6,318,742	6,321,487	2,746	6	233.7	268.8
Gorai.011G160100	PF01471	*-*	-	Chr11	29,253,663	29,256,718	3,056	7	221.1	233
Gorai.007G350700	PF01485	*ARI7*	Probable E3 ubiquitin-protein ligase ARI7	Chr07	58,079,030	58,087,989	8,960	17	153.4	392.3
Gorai.010G007500	PF01565	*CKX5*	Cytokinin dehydrogenase 5	Chr10	477,052	481,619	4,568	5	429	605.8
Gorai.006G084300	PF01657	*CRK26*	Cysteine-rich receptor-like protein kinase 26	Chr06	31,770,489	31,773,653	3,165	7	352.3	116.5
Gorai.001G121500	PF01754	*SAP3*	Zinc finger A20 and AN1 domain-containing stress-associated protein 3	Chr01	14,830,583	14,831,995	1,413	2	564.5	284
Gorai.002G234400	PF02045	*NFYA10*	Nuclear transcription factor Y subunit A-10	Chr02	59,489,436	59,493,917	4,482	6	278.8	561.8
Gorai.012G141600	PF02152	*FOLB1*	Dihydroneopterin aldolase 1	Chr12	31,505,796	31,507,873	2,078	3	261.3	647
Gorai.007G350900	PF02182	*SUVH1*	Histone-lysine N-methyltransferase, H3 lysine-9 specific SUVH1	Chr07	58,110,934	58,115,639	4,706	2	1,484.00	1,738.00
Gorai.010G012000	PF02365	*NAC053*	NAC domain-containing protein 53	Chr10	903,218	907,562	4,345	7	221.6	465.7
Gorai.010G008100	PF02458	*HHT1*	Omega-hydroxypalmitate O-feruloyl transferase	Chr10	538,869	541,376	2,508	2	874.5	759
Gorai.011G158900	PF02636	*SPAC25A8*.*03c*	-DH dehydrogenase [ubiquinone] complex I, assembly factor 7 homolog	Chr11	28,720,032	28,729,435	9,404	14	129	584.5
Gorai.001G088800	PF02704	*RSI-1*	Protein RSI-1	Chr01	9,592,197	9,594,141	1,945	3	248.7	599.5
Gorai.011G141100	PF02776	*-*	Acetolactate synthase 3, chloroplastic	Chr11	22,244,554	22,246,858	2,305	1	2,305.00	intronless
Gorai.002G241400	PF02922	*SBEI*	1,4-alpha-glucan-branching enzyme 1, chloroplastic/amyloplastic	Chr02	60,478,914	60,489,748	10,835	23	135.5	336.9
Gorai.007G347600	PF02970	*TFCA*	Tubulin-folding cofactor A	Chr07	57,776,551	57,778,600	2,050	4	197.3	420.3
Gorai.003G137500	PF03081	*EXO70A1*	Exocyst complex component EXO70A1	Chr03	39,661,653	39,663,784	2,132	1	2,132.00	intronless
Gorai.002G235800	PF03168	*YLS9*	Protein YLS9	Chr02	59,628,429	59,630,118	1,690	1	1,690.00	intronless
Gorai.011G135200	PF03405	*-*	Stearoyl-[acyl-carrier-protein] 9-desaturase, chloroplastic	Chr11	20,394,798	20,400,526	5,729	3	459.7	2,175.00
Gorai.011G137800	PF04012	*IM30*	Membrane-associated 30 kDa protein, chloroplastic	Chr11	21,162,830	21,169,837	7,008	12	160.6	461.9
Gorai.001G121400	PF04212	*SKD1*	Protein SUPPRESSOR OF K(+) TRANSPORT GROWTH DEFECT 1	Chr01	14,812,123	14,817,289	5,167	8	234.8	469.9
Gorai.007G348700	PF04674	*EXL6*	Protein EXORDIUM-like 6	Chr07	57,893,387	57,894,787	1,401	2	572.5	256
Gorai.009G367100	PF04690	*YAB5*	Axial regulator YABBY 5	Chr09	49,185,118	49,189,637	4,520	8	137.6	488.4
Gorai.006G024500	PF04981	*NMD3*	60S ribosomal export protein NMD3	Chr06	6,301,408	6,304,180	2,773	2	1,041.50	690
Gorai.011G142800	PF05419	*GUN4*	Tetrapyrrole-binding protein, chloroplastic	Chr11	22,533,055	22,534,264	1,210	1	1,210.00	intronless
Gorai.006G067100	PF05553	*-*	-	Chr06	26,559,871	26,560,732	862	1	862	intronless
Gorai.002G237800	PF05577	*PRCP*	Lysosomal Pro-X carboxypeptidase	Chr02	60,091,336	60,098,128	6,793	9	214.7	585.1
Gorai.006G032900	PF05691	*RFS5*	Probable galactinol—sucrose galactosyltransferase 5	Chr06	8,508,771	8,511,521	2,751	4	619.5	91
Gorai.011G141200	PF05695	*ycF2*:*3 -A*	Protein YcF2:3	Chr11	22,256,670	22,258,052	1,383	2	201	981
Gorai.011G142600	PF05773	*GCN2*	Probable serine/threonine-protein ki-se GCN2	Chr11	22,507,944	22,525,343	17,400	28	140.9	498.3
Gorai.007G347500	PF06219	*-*	-	Chr07	57,772,596	57,776,558	3,963	4	506	643.7
Gorai.010G007700	PF07526	*BLH11*	BEL1-like homeodomain protein 11	Chr10	487,521	490,937	3,417	4	395.8	611.3
Gorai.006G024600	PF07714	*At5g15080*	Probable receptor-like protein kinase At5g15080	Chr06	6,313,178	6,317,740	4,563	6	347.3	487.2
Gorai.007G349400	PF07714	*At3g07070*	Serine/threonine-protein kinase At3g07070	Chr07	57,951,540	57,955,486	3,947	5	365.6	529.8
Gorai.009G347700	PF07797	*-*	-	Chr09	43,080,219	43,081,973	1,755	3	376	118.5
Gorai.002G241300	PF07992	*AFRR*	Monodehydroascorbate reductase	Chr02	60,463,573	60,466,896	3,324	10	177.9	171.7
Gorai.007G353300	PF08159	*nol10*	Nucleolar protein 10	Chr07	58,413,029	58,420,998	7,970	16	169.8	350.3
Gorai.002G235300	PF08263	*TMK3*	Receptor-like kinase TMK3	Chr02	59,576,586	59,580,461	3,876	3	1,164.30	190
Gorai.010G010100	PF08263	*At2g16250*	Probable LRR receptor-like serine/threonine-protein kinase At2g16250	Chr10	751,818	757,264	5,447	5	725	455.5
Gorai.011G162100	PF08263	*RCH2*	Receptor-like protein kinase 2	Chr11	30,378,807	30,382,872	4,066	2	1,983.50	99
Gorai.009G367300	PF08523	*MBF1B*	Multiprotein-bridging factor 1b	Chr09	49,207,335	49,209,624	2,290	4	227.5	460
Gorai.011G137400	PF09247	*TAF1*	Transcription initiation factor TFIID subunit 1	Chr11	21,135,957	21,154,677	18,721	21	320.1	600
Gorai.006G099800	PF09405	*-*	-	Chr06	34,021,045	34,027,401	6,357	12	260	294.3
Gorai.007G353400	PF09713	*-*	-	Chr07	58,422,969	58,430,414	7,446	9	161.3	735.6
Gorai.011G170500	PF10153	*efg1*	rRNA processing protein efg1	Chr11	37,394,490	37,397,919	3,430	8	189.6	254.9
Gorai.009G366500	PF10517	*At5g54830*	Cytochrome b561, DM13 and DOMON domain-containing protein At5g54830	Chr09	49,101,413	49,107,783	6,371	2	1,677.00	3,017.00
Gorai.002G235400	PF11571	*MED27*	Mediator of R- polymerase II transcription subunit 27	Chr02	59,598,071	59,602,249	4,179	7	274.9	375.8
Gorai.006G032700	PF12530	*RST1*	Protein RST1	Chr06	8,479,478	8,494,554	15,077	25	231.6	386.9
Gorai.007G221800	PF12767	*-*	-	Chr07	25,772,747	25,774,873	2,127	2	889.5	348
Gorai.003G137600	PF12796	*At5g02620*	Ankyrin repeat-containing protein At5g02620	Chr03	39,671,615	39,673,455	1,841	3	542	107.5
Gorai.001G122200	PF12937	*At1g67190*	F-box/LRR-repeat protein At1g67190	Chr01	15,251,697	15,256,244	4,548	3	773	1,114.50
Gorai.001G121300	PF13041	*PCMP-H60*	Pentatricopeptide repeat-containing protein At2g27610	Chr01	14,807,577	14,810,668	3,092	3	982	73
Gorai.006G023300	PF13041	*PCMP-E79*	Putative pentatricopeptide repeat-containing protein At3g28640	Chr06	6,089,690	6,092,140	2,451	1	2,451.00	intronless
Gorai.010G009700	PF13041	*At1g19525*	Pentatricopeptide repeat-containing protein At1g19525	Chr10	712,070	715,930	3,861	3	930.7	534.5
Gorai.007G353200	PF13419	*Nanp*	N-acylneurami-te-9-phosphatase	Chr07	58,396,486	58,400,809	4,324	6	219.8	601
Gorai.011G142500	PF13540	*ACR4*	Serine/threonine-protein kinase-like protein ACR4	Chr11	22,503,343	22,506,977	3,635	1	3,616.00	intronless
Gorai.006G099500	PF13637	*Ank2*	Ankyrin-2	Chr06	33,992,982	33,995,636	2,655	4	362.3	402
Gorai.011G158400	PF13833	*CML22*	Probable calcium-binding protein CML22	Chr11	28,182,638	28,185,225	2,588	5	256	327
Gorai.002G231400	PF13837	*-*	-	Chr02	59,082,050	59,084,231	2,182	1	2,182.00	intronless
Gorai.002G231500	PF13837	*GT-2*	Trihelix transcription factor GT-2	Chr02	59,100,465	59,102,613	2,149	2	1,025.00	99
Gorai.002G234500	PF13855	*At1g74360*	Probable LRR receptor-like serine/threonine-protein kinase At1g74360	Chr02	59,494,567	59,496,769	2,203	3	695.3	58.5
Gorai.010G012100	PF13867	*-*	-	Chr10	917,545	920,366	2,822	6	221.5	298.6
Gorai.006G032800	PF14259	*At3g27700*	Zinc finger CCCH domain-containing protein 41	Chr06	8,495,295	8,503,847	8,553	6	546.8	1,054.40
Gorai.011G162300	PF14291	*-*	-	Chr11	30,391,532	30,394,378	2,847	5	207	453
Gorai.009G374700	PF14929	*-*	-	Chr09	50,940,936	50,945,978	5,043	9	230	371.6

### Gene mining, protein characterization and Gene Ontology (GO) functional annotations of the mined genes

We conducted a blast search at regions up and down stream of 20 Kb of each SSR location using the total 846 SSR markers sequences that were extracted from D_5_ genome. 2,496 genes were identified. The genes were mapped in all the chromosomes the chromosome with the highest number of genes was Chr09 with 316 genes followed by Chr11with 257 genes while Chr02 had the least number of genes with only 48 genes. The genes were characterized for their physiochemical properties. The grand average hydropathy (GRAVY) values ranged between -2.335 and 1.654, their molecular weight ranged between 5.935 and 437.729 kDa, their charge ranged from -170.5 to 66 while the Isoelectric Point (pI) ranged from 3.435 to 12.839, there were 2049 genes that were hydrophilic while only 446 were hydrophobic as shown by their GRAVY values (S4 Table).

**Fig 2:**
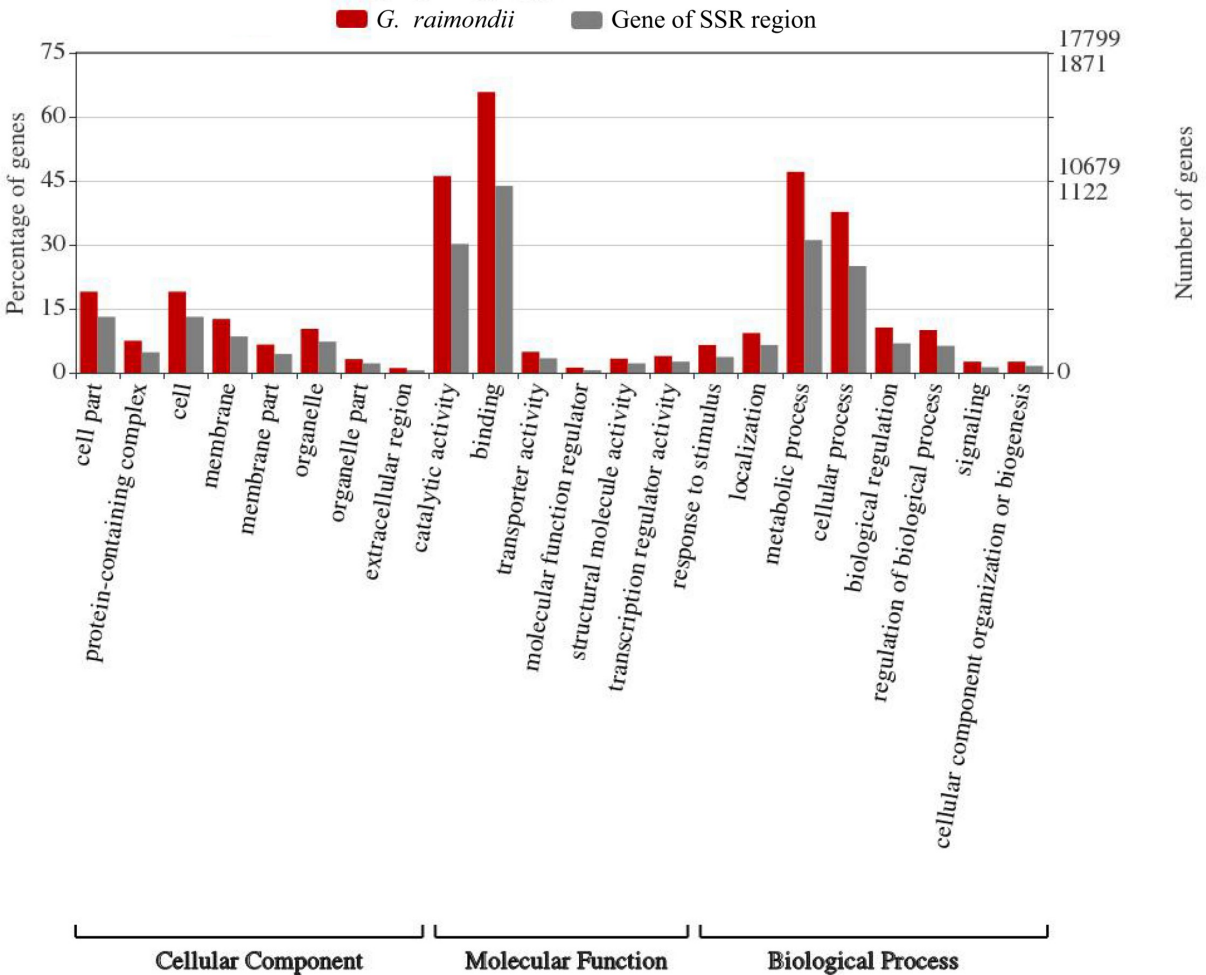
GO annotation results from the mined genes, the highest being cellular component with 9 functions.

From the GO blast analysis, all the three GO terms were detected, in which the highest being the cellular component with 9 functions, while the least was the molecular component with 6 functions (Fig 2). In cellular component (CC), the following genes were found to harbor critical functions, *Gorai*.*009G374600*, *Gorai*.*012G141400*, *Gorai*.*007G347200*, *Gorai*.*001G121600*, *Gorai*.*011G137100*, *Gorai*.*010G012200*, *Gorai*.*011G141200*, *Gorai*.*007G353300* and *Gorai*.*007G221800*. The CC functions detected were; nucleus (GO: 0005634), chloroplast (GO: 0009507), membrane (GO: 0016020), integral to membrane (GO: 0016021), integral to Golgi membrane (GO: 0030173) and SAGA-type complex (GO: 0070461).

The integrity of cell membrane and cell membranous is significant for normal functioning of the plant, when plants are exposed to any form of stress, the excessive production of reactive oxygen species (ROS), do degrades the cell membrane thus affecting the normal osmotic balance within the cell, which eventually lead to cell death [41,42]. In molecular functions (MF), 60 genes were found to be involved, with 27 different molecular functions, such as transferase activity, transferring phosphorus-containing groups (GO: 0016772), calcium ion binding (GO: 0005509), protein binding (GO: 0005515), transferase activity, transferring acyl groups other than amino-acyl groups among others (GO: 0016747), among others. In the determination of a gene with higher contributory role in cotton fiber development, a gene in ligon lintless-1 gene (*Li*_*1*_ gene) was found to harbor various molecular functions such as transferring acyl groups other than amino-acyl groups among others (GO: 0016747), which has been found to play an important role in cotton fiber elongation [43].

Finally, the GO functions detected to be involved in biological processes were 15, which included functions such as oxidation-reduction process (GO: 0055114), translational elongation (GO: 0006414), folic acid-containing compound metabolic process (GO: 0006760), response to light stimulus (GO: 0009416), regulation of transcription, DNA-dependent (GO: 0006355), microtubule-based movement (GO: 0007018) among others. Oxidation-reduction process, is important in plants responses to stress conditions [44], thus, the detection of this biological function among the genes obtained within the SDRs perhaps indicates that, these genes could be having a stress responsiveness functions in enhancing plants survival under abiotic stress conditions. Detailed information on the GO functions and the genes involved are summarized in (S5 Table). In the identification and characterization of the late embryogenesis abundant proteins (LEA) in cotton, Magwanga et al [1], found that integral to membrane (GO: 0016021), was detected for over 95% of the *LEA* genes, and this he postulated to have a functional role in maintaining the cell membrane integrity. Moreover, in the analysis of the genes which could been introgressed into the backcross population, BC_2_F_2_ developed from *G*. *tomentosum* a drought and salt resistant donor parent and *G*. *hirsutum* a high yielding tetraploid cotton but more susceptible to various forms of abiotic stress [18], revealed several GO functions, some which have been detected for the genes obtained within the SDRs in this study, an indication that these genes could be playing an important role in the plant.

### Analysis of the structure of the genes within the SDR

We undertook to analyze the structures of the genes found within the segregation distortion regions as obtained for chr1, chr2, chr6, chr7, chr9, chr10 and chr11 with 9, 35, 16, 33, 10, 12 and 30 genes, respectively. Out of all the genes within the SDRs, 22 were intronless, of significant were *Gorai*.*011G142800* (Tetrapyrrole-binding protein, chloroplastic), *Gorai*.*002G235800* (Protein *YLS9*), *Gorai*.*011G141100* (Acetolactate synthase 3, chloroplastic), *Gorai*.*011G158300* (Receptor like protein kinase S.2), *Gorai*.*007G347800* (Cytochrome P450 89A2), *Gorai*.*011G142500* (Serine/threonine-protein kinase-like protein ACR4), *Gorai*.*001G121600* (Beta-1,6-galactosyltransferase *GALT29A*) and *Gorai*.*006G023300* (Putative pentatricopeptide repeat-containing protein *At3g28640*) (Table 2). In all the genes within the SDRs, 23 genes were classified as genes of unknown domain, accounting for 16% of all the genes mined within the various SDRs across the seven (7) chromosomes, being the remaining genes were from different domains. The dominant domain among the remaining 123 genes was the P450; Cytochrome P450 (PF00067) with six (6) genes which were Gorai.001G088900 (Cytochrome P450 81D1), Gorai.001G089000 (Cytochrome P450 81E8), Gorai.002G241100 (Cytochrome P450 94C1), Gorai.006G081800 (Cytochrome P450 CYP736A12), Gorai.007G347700 (Cytochrome P450 89A2) and Gorai.007G347800 (Cytochrome P450 89A2). Cytochromes P450 (CYPs)are proteins of the superfamily containing heme as a cofactor), therefore, they are hemoproteins [45]. The cytochromes (CYPs) use a variety of small and large molecules as substrates) in enzymatic reactions. They are the terminal oxidase enzymes in electron transfer chains, broadly categorized as P450-containing systems The term "P450" is derived from the spectrophotometric peak at the wavelength of the absorption maximum of the enzyme (450 nm) when it is in the reduced state and combined with carbon (II) oxide. In plants, CYPs are involved in numerous biosynthetic reactions, which leads to plant hormones production, secondary metabolites synthesis, fatty acid conjugation, lignification of various plant tissues, and production of various defensive compounds [46]. Plant cytochrome P450 genes make up 1% of the plant genes as per the annotations of plant genome. The number and diversity of these genes is believed to trigger numerous bioactive compounds [47]. The detection of these genes within the SDR could explain the significance of these regions in the evolution of new functional transcriptome within the plants.

### Collinearity analysis between the genetic map and the physical map reference genome, *G*. *raimondii* (D_5_)

**Fig 3:**
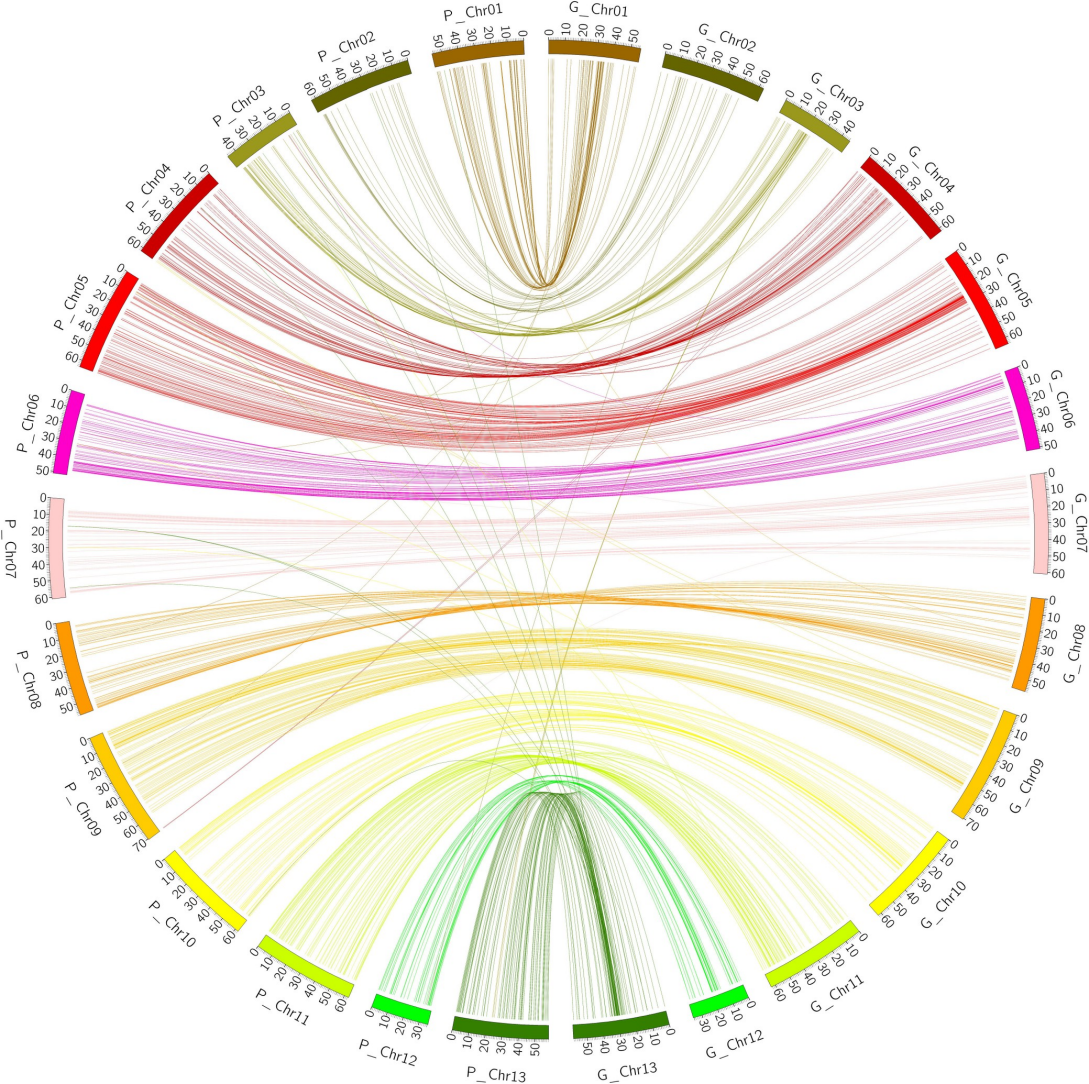
Collinearity between genetic and physical map of D_5_. The different colors represent the various syntenic block regions in the chromosomes.

We performed Collinearity analysis between the constructed genetic map of *G*. *thurber*i and *G*. *trilobum* with a reference to *G*. *raimondii* physical map. All the SSR markers full sequences were used to do blast search against the physical genomic map of *G*. *raimondii*; and the matching sites were extracted from blast result for collinearity analysis. After removal of redundant markers 846 SSR markers located in genetic linkage map produced 869 loci, which translated to 95.9% of the mapped markers showed consistency between two maps (Fig 3); however there were 36 markers that were in non-conformity to the physical map of the reference genome (Table 3). There were six (6) inversions noted on Chr02, Chr03, Chr07, and Chr13, and also four translocations in Chr03, Chr08, and Chr09 (Table 4), the map developed was of high resolution. Comparison of genetic and physical map is important in confirming the order of genetic markers, using the information from sequence-based physical maps and also to support the genetic-marker order [48]. The collinearity analysis conducted between our genetic map and the physical map with the reference genome being *G*. *raimondii* indicated good collinearity between the chromosomes in the genetic map and the physical map; it also confirms the accuracy of the genetic map.

**Table 3:** Marker inconformity between the genetic and physical map.

Markers	Genetic location (cM)		Physical location (bp)		
	Linkage group	Location	Chromosome	Start	End
SWU18284	G_Chr01	52.019	P_Chr09	22316264	22316364
SWU15054	G_Chr01	61.469	P_Chr06	23180998	23181098
SWU16907	G_Chr01	72.737	P_Chr08	14869954	14870054
SWU12734	G_Chr02	15.036	P_Chr03	39667891	39667991
SWU21545	G_Chr02	19.215	P_Chr12	31489218	31489318
SWU15145	G_Chr03	2.45	P_Chr06	31792020	31792120
SWU14306	G_Chr03	32.028	P_Chr05	32177040	32177140
SWU22022	G_Chr03	32.614	P_Chr13	22964679	22964779
SWU22040	G_Chr03	32.803	P_Chr13	24293706	24293806
SWU22028	G_Chr03	33.245	P_Chr13	23380707	23380807
SWU18938	G_Chr04	0	P_Chr09	70170969	70171069
SWU18954	G_Chr04	3.422	P_Chr09	70478586	70478686
SWU12135	G_Chr06	0	P_Chr03	7642054	7642154
SWU15193	G_Chr07	28.58	P_Chr06	34002005	34002105
SWU14289	G_Chr07	33.571	P_Chr05	30830808	30830908
SWU19619	G_Chr07	41.198	P_Chr10	50389834	50389934
SWU10330	G_Chr08	36.173	P_Chr01	15267145	15267245
SWU13865	G_Chr09	0	P_Chr04	61146573	61146673
SWU13887	G_Chr09	12.515	P_Chr04	62008452	62008552
SWU15131	G_Chr10	38.761	P_Chr06	31048453	31048553
SWU16204	G_Chr10	73.246	P_Chr07	30300267	30300367
SWU14046	G_Chr11	42.846	P_Chr05	8058887	8058987
SWU12579	G_Chr11	51.861	P_Chr03	33127023	33127123
SWU16010	G_Chr13	19.276	P_Chr07	16783626	16783726
SWU16010	G_Chr13	19.276	P_Chr07	16783691	16783791
SWU19829	G_Chr13	38.499	P_Chr10	61306040	61306140
SWU16525	G_Chr13	44.267	P_Chr07	55378850	55378950
SWU16010	G_Chr13	47.694	P_Chr07	16783626	16783726
SWU16010	G_Chr13	47.694	P_Chr07	16783691	16783791
SWU12058	G_Chr13	49.895	P_Chr03	4567547	4567647
SWU11359	G_Chr13	52.381	P_Chr02	28492364	28492464
SWU13955	G_Chr13	55.048	P_Chr05	3003174	3003274
SWU12482	G_Chr13	55.598	P_Chr03	27549570	27549670
SWU10804	G_Chr13	57.636	P_Chr01	50739519	50739619
SWU13263	G_Chr13	58.021	P_Chr04	18325212	18325312
SWU13263	G_Chr13	58.021	P_Chr04	18325260	18325360

**Table 4:** Chromosomes showing inversion and translocation between the genetic and physical map of D_5_.

Chromosome No.	Markers	Location of Genetics (cM)	Location of Physical map (Mb)	Event
Chr02	SWU11559-SWU11210	6.558–8.012	15.95–50.80	inversions
Chr03	SWU12367-SWU22028	31.53–33.245	19.33–23.38	translocations
Chr03	SWU12731-SWU12733	24.321–24.575	39.56–39.87	translocations
Chr03	SWU12611-SWU12621	28.579–28.597	34.51–34.81	inversions
Chr05	SWU14526-SWU14481	40.808–41.701	48.23–51.89	inversions
Chr07	SWU16069-SWU16057	27.244–27.499	19.34–20.05	inversions
Chr08	SWU16899-SWU16916	66.435–67.516	14.37–15.76	translocations
Chr09	SWU18603-SWU18528	86.688–87.121	38.71–46.91	translocations
Chr13	SWU21880-SWU21921	54.134–54.181	8.43–11.56	inversions
Chr13	SWU22169-SWU22142	51.086–51.253	35.16–36.79	inversions

### Collinearity analysis of genetic map to that of the physical map (Dt) for *G*. *hirsutum* (GhDt) and *G*. *barbadense* (GbDt)

**Fig 4:**
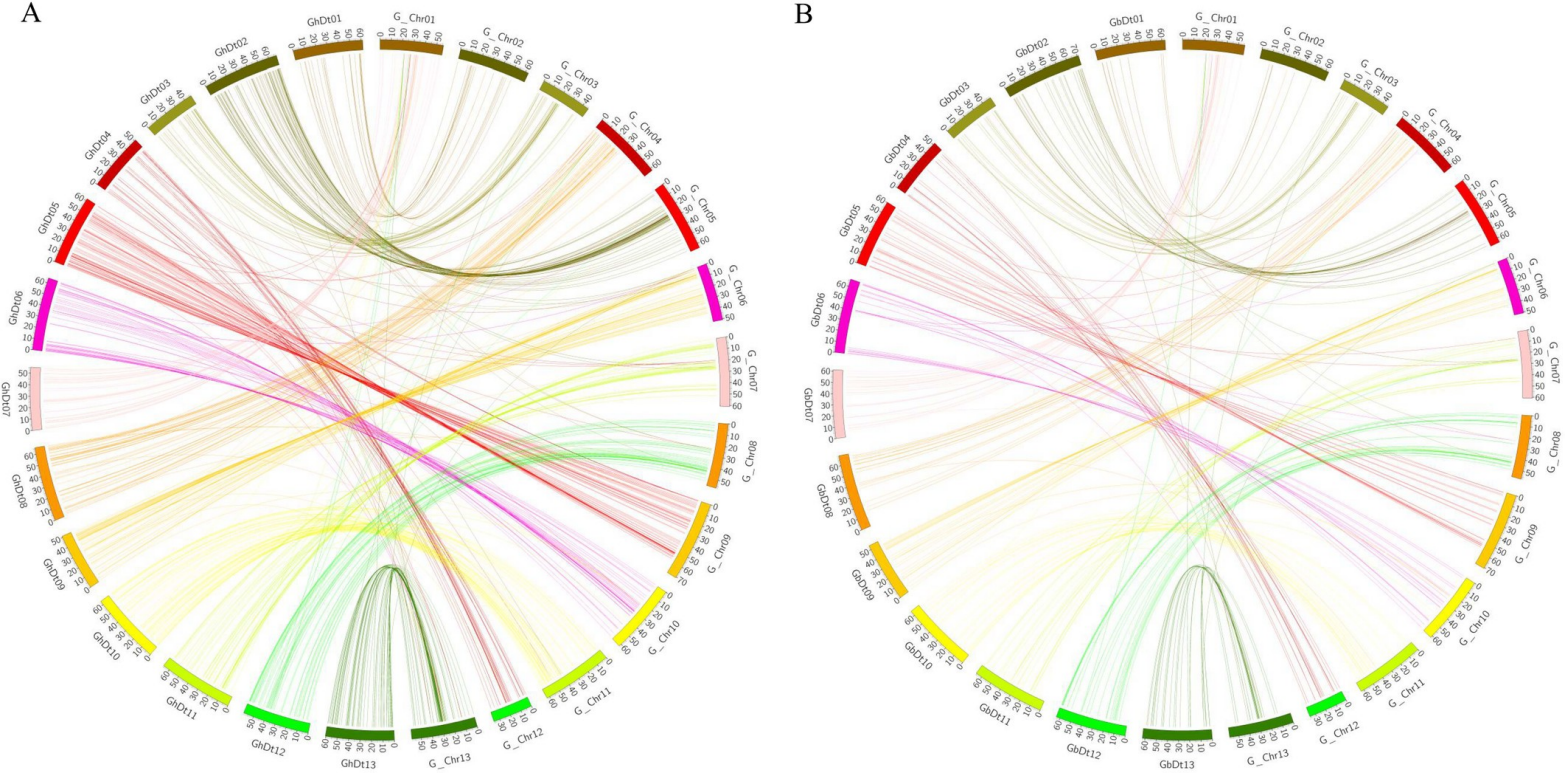
Collinearity analysis. (A) Analysis between genetic map and physical map of GhDt while (B). Analysis between genetic map and physical map of GbDt.

A blast search was conducted by using 846 SSR markers from the genetic map and was screened on the Dt-sub genome of *G*. *hirsutum* and *G*. *barbadense*, out of the total markers 745 and 337 markers were aligned to the assembly genome of *G*. *hirsutum* (GhDt) and *G*. *barbadense* (GbDt) respectively. 16% of marker in GhDt and 15% of markers in GbDt showed consistency between two maps; however 85% of markers in both sub genomes were in non-conformity between genetic and physical map (Fig 4A, Fig 4B, S6 Table and S7 Table). From the results obtained on the two collinearity analysis, showed that there was a closer relationship between the genetic map and physical map of GhDt than GbDt, this is clearly shown by higher number of markers that were in conformity with GhDt rather than GbDt, this lead to formation of better syntenic blocks between the genetic and physical map of GhDt. Good syntenic block formation was however observed between chromosome 3 in both the two sub genomes. This could possibly mean that more genes have been introgressed from the two wild cotton species into *G*. *hirsutum* rather than to *G*. *barbadense*, from previous studies on genes introgression within *G*. *hirsutum*, a higher percentage of the introgression observed (43.7%) was accounted for by wild accessions, as compared to improved accessions (18.4%) whereas within *G*. *barbadense*, 33.1% of the introgression was accounted for by wild accessions, and only 27.1% of the introgression was accounted for by improved accessions, thus the wild accessions accounted for more introgression in *G*. *hirsutum* than in *G*. *barbadense* [49]. The results obtained are in agreement to the earlier reports, in which fibre quality traits such as, high fibre length have been found to be introgressed into *G*. *hirsutum* from its wild progenitors of the D genome, *G*. *thurberi* [5]. Moreover, *G*. *hirsutum* and *G*. *barbadense* are said to have a common ancestry however interference by human activities and abiotic factors have made them to evolve different agronomic traits [50]. However, their genomic sequences have made it possible to study the divergence and comparative analysis of the two species.

### RNA sequence data analysis and RT-qPCR validation of genes in the SDR regions

**Fig 5:**
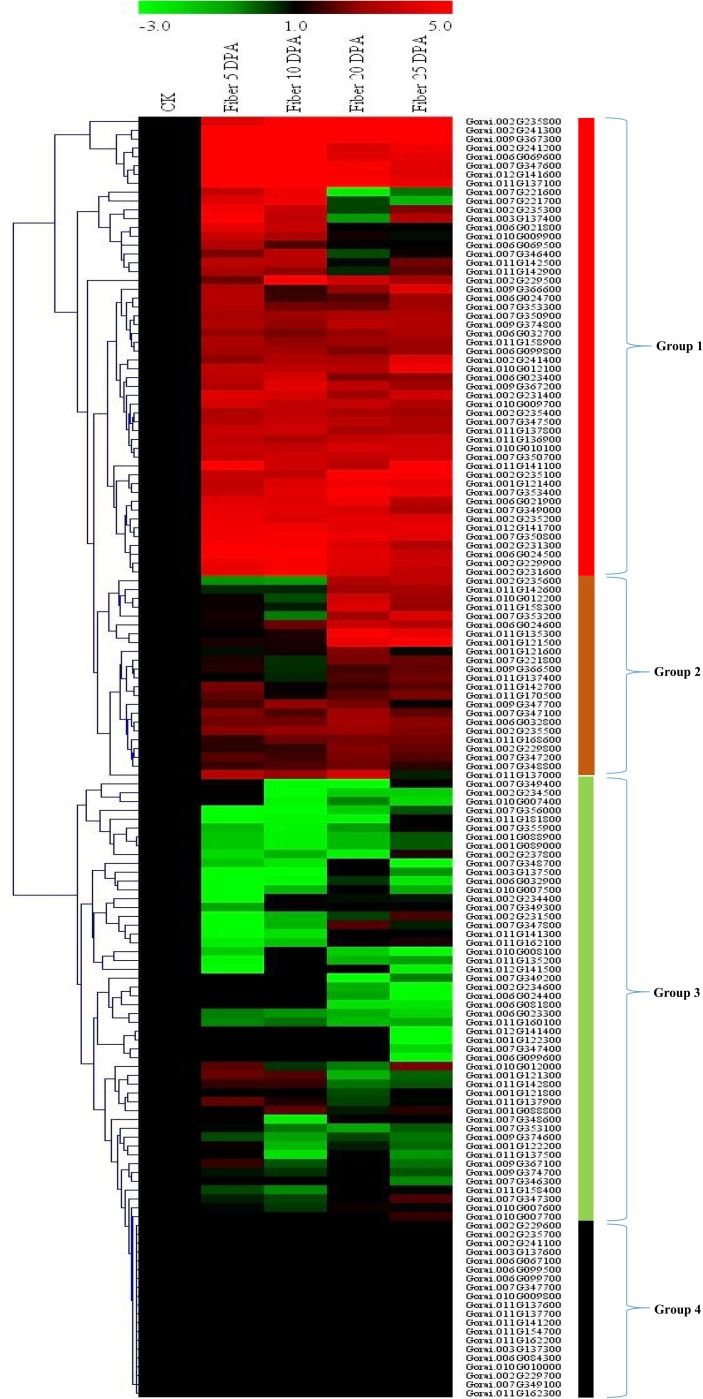
Heat map for the RNA expression seq. for the mined genes at the SDR regions in relation to fiber development. The heat map was visualized using Mev.exe program (Showed by log 2 values). (i) Red-up regulated, green-down regulated and black- no expression. DPA: day post anthesis.

**Fig 6:**
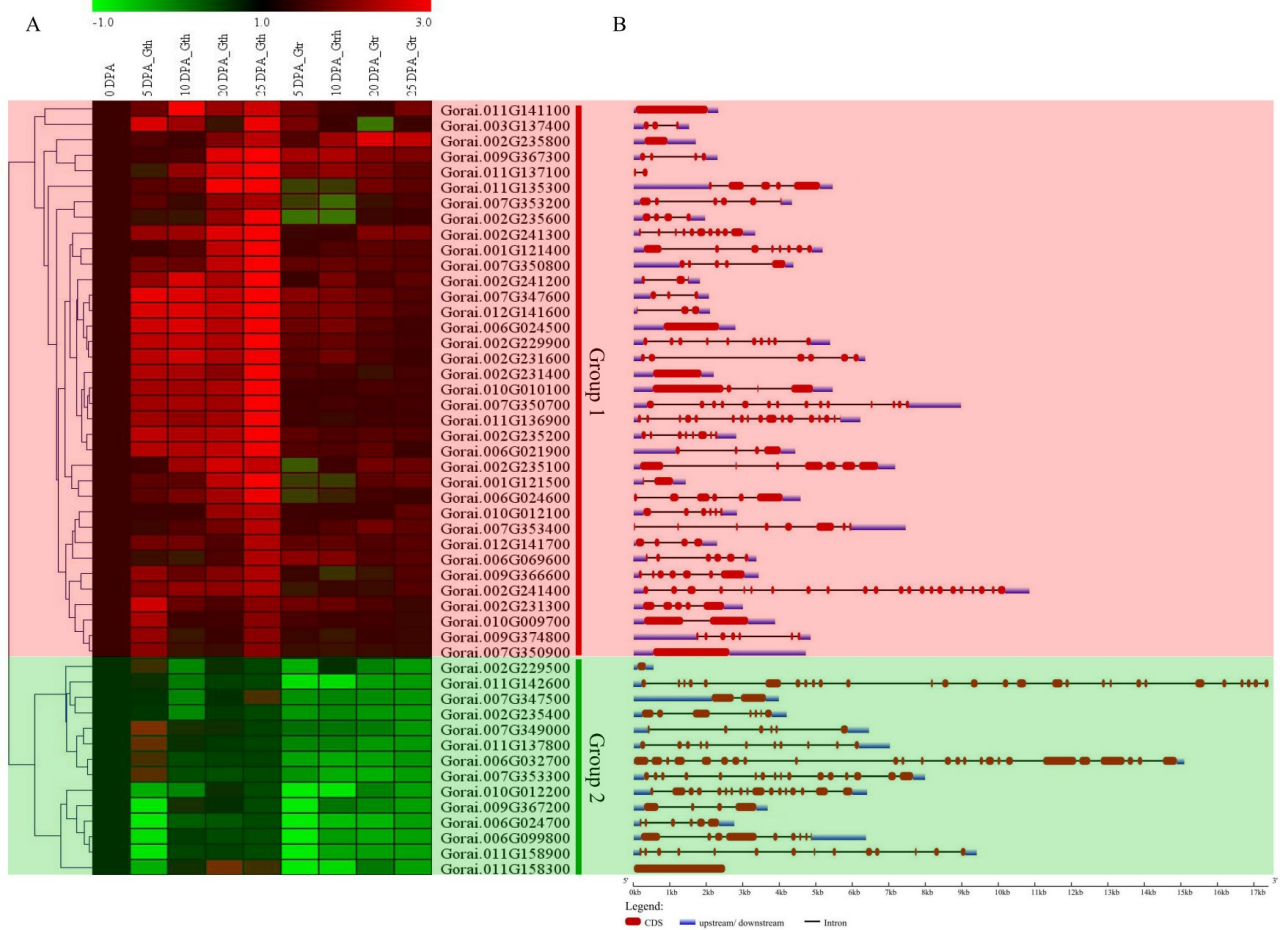
RT-qPCR validation of the selected genes. **(A): Heat map for the 50 highly upregulated genes as per the RNA seq**. The heat map was visualized using Mev.exe program (Showed by log 2 values). (i) Red-up regulated, green-down regulated and black- no expression. DPA: day post anthesis (B): Gene structure analysis of the 50 selected genes. Abbreviation: DPA: day post anthesis; Gth: *Gossypium thurberi*; Gtr: *Gossypium trilobum*.

The RNA sequence data for the genes at the SDR were obtained using their homeolog genes of the upland cotton; *G*. *hirsutum* sequenced at different development stages cotton fiber 0, 5, 10, 20 and 25 day post anthesis (DPA) from cotton genome data base and analyzed. Based on the expression profile, the genes were categorized into four groups. Group 1 members were highly up regulated, though some were found to be only highly up regulated in some stages of fiber development. Group 2, exhibited differential expression, with some being up regulated while other were either down regulated or not expressed. Group 3, majority of the genes were down regulated, with only very few genes were either partially up regulated or not expressed. Lastly groups 4 were not expressed in all the various stages of fiber development (Fig 5). Furthermore, 30 highly upregulated genes later validated through RT-qPCR, at similar fiber development stages. Majority of the genes were highly inducted in *G*. *thurberi* as compared to *G*. *tribolum* an indication that *G*. *thurberi* had a higher potential of producing superior fibers compared to *G*. *trilobum* (Fig 6A). Similar findings have been previously reported in which *G*. *thurberi* has been found to have superior [19]. The expression pattern for the fifty (50) analysed genes through RT-qPCR analysis revealed that these genes could be playing an important role in various stages of cotton fiber development, for instance *Gorai*.*007G347600* (Tubulin-folding cofactor A), *Gorai*.*012G141600* (Dihydroneopterin aldolase 1), *Gorai*.*006G024500* (60S ribosomal export protein NMD3), *Gorai*.*002G229900* (Protein LST8 homolog) and *Gorai*.*002G235200* (Ribosome biogenesis protein NSA2 homolog) were highly upregulated at different stages of fiber development. Studies have been conducted to investigate the role of tubulin in cotton fiber development, the tubulin genes were found to be upregulated at specific stages of fiber development, the transcript α-tubulin genes *GhTua2/3* and *GhTua4* were found to be highly upregulated from 10 to 20 DPA, while *GhTua1* and *GhTua5* transcripts were highly upregulated from 0 DPA up to 14 DPA then registered a significant drop at 16 DPA with the onset of secondary wall synthesis [51–53]. For the results obtained in the RNA sequence and RT-qPCR analysis, we observed that the highly up regulated genes were mainly enzymes that performed catalytic activities. Similar results have been observed in maize where gene cluster was observed on five adjacent genes (Bx1–Bx5) that encode enzymes for successive steps in the biosynthesis of the cyclic hydroxamic acid 2,4-dihydroxy-1,4-benzoxazin-3-one [54]. We further analysed the gene structures of the 50 highly up regulated genes as per the RNA sequencing results, all the genes were disrupted by introns except six (6) genes, which were *Gorai*.*010G010100* (Probable LRR receptor-like serine/threonine-protein kinase At2g16250), *Gorai*.*011G141100* (Acetolactate synthase 3, chloroplastic), *Gorai*.*002G231400* (uncharacterized gene), *Gorai*.*011G158300* (Receptor like protein kinase S.2), *Gorai*.*007G350900* (Histone-lysine N-methyltransferase, H3 lysine-9 specific SUVH1) and *Gorai*.*006G024500* (60S ribosomal export protein NMD3) (Fig 6B). Several stress responsive genes have been found to be heavily laden with introns, such as the *LEA* genes [55], cyclin dependent kinase (*CDK*) genes [56], G protein coupled receptors (*GPCRs*) [57] among others.

## Discussion

Construction of genetic maps has become increasingly important in the understanding of marker- assisted selection (MAS) in plants and its efficiency in gene mapping. The use of SSR markers in the construction of genetic maps have a great significance since these markers are abundant and have high levels of transferability, low levels of intra-locus, relative abundance and good genome coverage [58]. In the development of our genetic map we employed the use of EST-SSR (eSSRs) SWU mono markers, ESR-SSR markers have been used in the construction of several genetic maps in cotton.[59–61] The ESSRs are based on expressed sequences and are conserved across cotton species and other closely related plant species.

Wild cotton has been known to have both advantageous and disadvantageous traits, therefore the development of genetic maps of interspecific and intraspecific crosses aids in the introgression of advantageous alleles into the already cultivated cultivars. We developed a genetic map, from an interspecific cross between two wild cotton species in diploid cotton in the D genome. So far, fewer genetic maps developed from the diploid D genome have been reported. The constructed genetic map had a total length of 1,012.458 cM with an average distance between the loci of 1.193 cM, a total of 849 markers loci were used in the map development. The map developed had a much higher genome coverage compared to earlier developed maps from the diploid genomes, for instance, a map between *G*. *arboreum* and *G*. *herbaceum* had a total length of 1,109 cM with an average marker distance of 7.92 cM between loci [59]. Although the map size was relatively smaller compared to dense genetic maps developed, the average distance between two marker loci (1.193 cM) was smaller compared to previously developed maps, this indicates that the map is suitable for analysis of quantitative trait loci (QTLs) and gene mining [62]. The genetic map between the two sister species *G*. *thurberi* and *G*. *trilobum* is the second to have been developed from wild species in the D genome, the first map was developed from an interspecific cross between *G*. *davidsonii* and *G*. *klotzschianum*. However several maps have been developed using diploid cotton from A genomes.

Segregation distortion (SD) is the deviation observed on genotypic frequencies from expected Mendelian ratios [63]. This phenomenon has been reported in other plants such as Maize [64], Barley [65] and Potatoes [66]. From our genetic map we noted segregation distortion loci (SDLs) in all the chromosomes; however they were unevenly distributed within the 13 chromosomes. Chr07 and Chr11 had the highest number of SDLs with 23 distorted loci each. Similar results were recorded on the genetic maps in *Gossypium spp* [61] Chr02, Chr07 and Chr11 [67], Chr07 (>50% of the loci were distorted). Some SDL were clustered in specific regions on the chromosome these regions were designated as the segregation distortion regions (SDRs). The largest SDRs were observed on Chr02, Chr06, Chr07-2 and Chr1. The largest SDR’s were skewed towards the heterozygous, similar results were also recorded from previous studies by Liang et al [38]. The skewness towards heterozygosity could be due to the genetic loci expressing themselves at different times leading to gametophytic and zygotic selection. Results from earlier constructed genetic map in cotton showed that larger SDRs were located on Chr02 [39], Chr02 had 3 SDRs (>50% of loci were distorted); [68], Chr02, Chr16 and Chr18 [69]. Most interestingly we also noted that in most of these genetic maps Chr02 had fewer number of marker loci. SDRs exhibiting similar patterns of distortion at the same chromosomal regions in several species-related populations could lead to the identification of common genetic factors causing these phenomena, [64]. From these results we concluded that Chr02 could be carrying vital genes that could be segregating around these SDRs and therefore making the flanking markers to segregate. Hence there is need to mine genes within these regions. The genes would help to ravel the issues of SDRs through the identification of important traits and genome wide association studies, for example Bovill et al [70] identified gene for crown rot resistance in wheat around the SDR, similar *Sr36* gene locus was detected in the SDR on chromosome 2B [71].

From the physiochemical properties of the genes mined we noted that the gravy values range were both positive and negative values, indicating that the proteins encoding the mined genes were both hydrophilic and hydrophobic in nature [72]. We noted that the identified genes were more hydrophilic in nature rather than hydrophobic, many genes contained proteins and enzymes related to metabolism and disease/defense; these proteins are mainly activated when in solution forms hence the occurrence of more hydrophilic genes than hydrophobic genes. We analyzed the genes located on the SDR to determine if they had role in segregation distortion of the flanking markers. We noted that within the SDR there were common gene domains; Cytochromes P450 (CYPs) appeared in almost all the SDRs with six members, the Myb-like DNA binding domain with four members and the Multi-protein bridging factor 1 domain with three members, these genes could probably have caused segregation of flanking markers. It could also be due to same gametophyte factors or unknown genes in a population segregating, thus exhibiting segregation distortion in the same chromosomal regions [73]. We further observed that the chromosomes with largest distortions had the highest number of genes; interestingly we noted that Chr02 had the least markers (21), with the shortest map size of 28.665 cM but had highest number of genes which were 35 genes. This implied that the genes located in these regions could have been segregating due to zygotic or gametophytic factors or other underlying factors hence there is need to do more research on these genes locate on the SDRs in chromosome 2. The expression analysis of the genes at the SDR region showed that some of the genes were involved in fibre development. The expression of genes in the SDR regions revealed that most of the genes that were up regulated were involved in enzyme catalytic activities; examples of these genes include Serine decarboxylase, Tetraketide alpha-pyrone reductase 2, Digalactosyldiacylglycerol synthase-1-chloroplastic, and Mediator of RNA polymerase II transcription subunit 27 among others.

The Cytochromes P450 (CYPs) were the dominant domain among the genes mined within the segregation regions (SDRs). This superfamily is among the largest group of enzymes in plants, they play vital role in a range of metabolic pathways. [74]. The name cytochrome is gotten from the spectral absorbance maximum, produced when carbon (II) oxide binds to the enzyme in its reduced state produced at 450 nm [75]. They have been found to function in all the eukaryotes. The two species *G*. *thurberi* and *G*. *trilobum* are also known to be resistance to soil-borne fungal pathogens, *Fusarium wilt* and *Verticillium wilt* respectively, enzymes play major function in various fungal metabolisms such as biochemical reactions, adaptation to hostile environment and detoxification of chemicals [76], and this could explain their higher number. In addition, *G*. *thurberi* also possess other beneficial agronomic traits, such as resistant to cotton bollworm and silver leaf whitefly, thus the reflection of the higher number of CYP, they play important role in both insects and plants, and they participate in a range of spectrum of plant toxins metabolized by insects and the defense compounds manufactured by plants. [77].

SDR has become a common feature in plants, and it is believed that the SDRs have significant effect on mapping and breeding applications. High level of distortions has been found in a number of plants, for instance in *Medicago sativa* L. 24% and 34% of markers have been found to be distorted in the mapping of the F_1_ generation, while in the F_2_ generation, very high level of distortion of 68% per linkage [78,79], similarly SD has also been observed in rice chromosome 9 in doubled haploid, recombinant inbred [64]. Segregation distortion is believed to be caused by a group of genetic elements near the centromere of chromosomes, and now been seen as a potentially powerful evolutionary force, has been observed in monocotyledons plants such as maize [80]. The detection of these genes provides further evidence of the significance role of the SDRs in plants.

## Conclusions

The use of genetic maps between wild cotton species has significance in identification of vital alleles with profound agronomic benefits that could be introgressed into elite cotton cultivar. Cotton farming is facing challenge emanating from environmental stresses such as cold, drought and salinity. The application of the two species would help molecular breeders in introgression of the identified vital genes into already cultivated cotton that were mined within the SSR regions. The two wild species in the D genome; *G*. *thurberi* as female parent and *G*. *trilobum* as the male parent were used in the construction of a fine genetic map, this map will provide a basic tool for researchers to conduct evaluation of QTLs and identification of novel genes along the SSR regions. The genetic map had a length of 1,012.458 cM with an average length between the loci of 1.193 cM. A total of 849 loci were successfully mapped in all the 13 chromosomes. Chromosome regions with obvious segregation distortion were identified in this map, approximately 16% of mapped markers showed distorted segregation in the F_2_progenies. 2,495 genes were mined within the SSR region and characterized on their physiochemical properties. We further analyzed the genes within the SDR region with an aim of identifying genes that could be segregating within the SDR, we noted that the common gene domain (Cytochromes P450 (CYPs) appeared in almost all the SDRs and it contained six members. Further analysis of this gene domain will enable understanding of the role they play in SDRs by future molecular breeders. The constructed linkage map will allow future breeders to identify the markers that linked to the trait of interest and use them in marker-assisted breeding program and genome wide studies.

## Ethical approval and consent to participate

No ethical nor consent to participate in this research was sought.

## Supporting information

S1 Table SWU marker sequences details. (XLSX)

S2 Table Details for primers used for RT-qPCR analysis. (DOCX)

S3 Table SWUmarkers and their allele scores. The male plant *G*. *trilobum*, the female plant *G*. *thurberi* and the heterozygous F_2_ progenies were scored as A, B, and H respectively. The missing data was designated as‘-’.Multi-allelic markers were named separately by primer name followed by the letters a, b, c, and d as a suffix.
(XLSX)

S4 Table Physiochemical properties of the proteins encoding the mined genes as obtained from the genetic map developed between the two wild cotton species of the D genome. (XLSX)

S5 Table Gene Ontology analysis of the genes obtained within the SDR regions. (DOCX)

S6 Table Markers inconformity between genetic map and the physical map *G*. *hirsutum* (GhDt). (DOCX)

S7 Table Markers inconformity between genetic map and the physical map of *G*. *barbadense* (GbDt). (DOCX)
