# Supplementary material for: SSR-Linkage map of interspecific populations derived from Gossypium trilobum and Gossypium thurberi and determination of genes harbored within the segregating distortion regions
Source: PLoS One. 2018 Nov 12;13(11):e0207271. doi: 10.1371/journal.pone.0207271 (PMC6231669; doi:10.1371/journal.pone.0207271)
Supplement: S2 Table — (DOCX) [file pone.0207271.s002.docx]

**Table S2**: Details for Primers used for RT-qPCR analysis

| Gene ID | Forward sequence | Reverse sequence |
| --- | --- | --- |
| Gorai.001G121400 | ATCCTTCCACCTCCTATTT | ACCTTCCTCTCCGAACTCA |
| Gorai.001G121500 | AAACCCAGCAACCCAAAAT | AGGAAACCGAAAACGACGA |
| Gorai.002G229500 | CTCTATGAGCGGGGTCCT | CATTGTTGAGATTGCGAC |
| Gorai.002G229900 | GAGGTTTTTCTTTGTTTTAT | TATCTCTTCACCACTTGTCA |
| Gorai.002G231300 | GCAACATTTCTCAATCAAC | GCAAACCAATCTAGCACAC |
| Gorai.002G231400 | ATCATCACCACCCTAACCC | AACTGAACATTTCCCTATT |
| Gorai.002G231600 | CTCACCTCTTCTTGCTCTT | TTGTTCACCTATTGTCTTT |
| Gorai.002G235100 | CCAGTGCTTTCTCGTTCAT | GTTGGCTCGGTCCTTCATA |
| Gorai.002G235200 | AGAAAAACAAAGCAATGGA | CTGAAGGACGGATGAAACG |
| Gorai.002G235400 | CCGTTGTGGCTTATGCTTG | TCGCCTTTTCTGGTCTGTG |
| Gorai.002G235600 | CGGTTTATGAGTTCCAGTT | GTTTTGTTGTTGCCTTTGC |
| Gorai.002G235800 | TCCATCCCTCCAACGTACC | TCATCAAGAAAACCGACCC |
| Gorai.002G241200 | TATTTGTTGGCTGTTTTGG | AGCTCTGTTATATCTTTGC |
| Gorai.002G241300 | GATTCACCTCCGATTCCAA | CACCAACACCGACAACAAC |
| Gorai.002G241400 | TGGTGGTGTGGGATTTGATT | CCTCCTGTTGGTTAGCGTGT |
| Gorai.003G137400 | CAGAAGGCTGCATTGAGGG | ATTGCTGGTGTTTGGTGAG |
| Gorai.006G021900 | AAACTGCTGTCTTACTCAG | AATCATCACCATACGAACC |
| Gorai.006G024500 | GATCGTGCACTCGGGTAC | GCTTCTTCTGGCGTTTTT |
| Gorai.006G024600 | GAAGCCGAAAGACCAGTG | CGAGTCCAAAATCAGAGA |
| Gorai.006G024700 | ACTGAAATCGGCAAGAAAG | CGGCAGAGGTAATAGCAAC |
| Gorai.006G032700 | TTTGCTGTTCAATTCCTAA | AAAGTTCCTGGTTTCCTCC |
| Gorai.006G069600 | AACATCGAGCATCTATCCT | GCCTCTCTATTTTCACACC |
| Gorai.006G099800 | ATCCTGGTATTGGGTCTCA | CACTGTTTTTCCCTTTGCT |
| Gorai.007G347500 | GGAATTTTTTATCGACACCG | GCTCATTACAAAGCCACCAG |
| Gorai.007G347600 | AGGATGATGGTCCCTGATTG | AACTTCGGTTATGGTGCTGC |
| Gorai.007G349000 | TTTTATCTACTGTCCCCACT | CTCTTGTCCACCAACATCCC |
| Gorai.007G350700 | ACAATAGAAAGACCAAAT | TGAGCAGTGACAAGTAAC |
| Gorai.007G350800 | TGCGAAACCAAAACCGAAG | GGATGGCAATGATAACCCG |
| Gorai.007G350900 | GACGACGATGGGCAAGACT | GAGGTGGGCACATAAAAGG |
| Gorai.007G353200 | CAGAGAAGCCAAATCCAAC | CAAAGCCAAGCATCACAAC |
| Gorai.007G353300 | AGCTTGAGGAGGGTGGACA | TAGGCTCGCAGAAGATTGG |
| Gorai.007G353400 | CTTGAAGAAGAGAATAGAG | GAATTAGAGATTGAGGAAA |
| Gorai.009G366600 | GTCTACCACTAAAGCACCCC | ATCTCATTGCCTTCAACTAC |
| Gorai.009G367200 | TATGAGAGAGATCGTTAGG | GAGAAAGAAAAAGGTAGAA |
| Gorai.009G367300 | TGGGAGCCCGTCGTTATTC | CTTGATGCCGCCCTATTCG |
| Gorai.009G374800 | TTAGAAGGCAGAAGGAAAGA | ATGATGAGATGAAGCAGGAA |
| Gorai.010G009700 | ACCCTAAACCCATTTCCCCT | TCCCATCATCCTCATCCTCA |
| Gorai.010G010100 | ACTTTGGATTTTTCGCTCG | AACTCCTCTGGTTTGGTCG |
| Gorai.010G012100 | ATCCGTCGAAAGAACAACT | CCTGAACAAATCCCACAAT |
| Gorai.010G012200 | TAACAGCCGAAGCAGAGAC | CCTAAAATGAGCAAAAGCA |
| Gorai.011G135300 | AATCGGATAAACGAAGTGGT | TAAAGAGGGGAATAAAGGAA |
| Gorai.011G136900 | TTCAGGAGGCTTGACATGG | GTAGTTTGAGGAGGGGGGC |
| Gorai.011G137100 | ACTTGGTGTGGTCCTTGG | TGAGATGTCGGCCTTTTT |
| Gorai.011G137800 | TTGTCTCTAACACACGGCTT | ACATTTCCCACCATCTCATT |
| Gorai.011G141100 | GCTCCTCCCCATTATTTCAT | CTTCACGTTCCAGGGCTTCC |
| Gorai.011G142600 | AGGGTCACCTCCACAAATC | TGGCAAGCACCTAACTAAG |
| Gorai.011G158300 | ACCCCCCAGTGGAAAAACC | CCCCGATGAAACCAACAAA |
| Gorai.011G158900 | CAAAAACAAACTGTAGGAG | GTGGAAGATTATCAAGCAC |
| Gorai.012G141600 | GAAATGGGATACCAACAGG | TCTACATCAACCAAGAACT |
| Gorai.012G141700 | ATTTCCCCGATTCCTCCCG | GTCACTGCGCCTTCCTTGC |
