# Supplementary material for: SSR-Linkage map of interspecific populations derived from Gossypium trilobum and Gossypium thurberi and determination of genes harbored within the segregating distortion regions
Source: PLoS One. 2018 Nov 12;13(11):e0207271. doi: 10.1371/journal.pone.0207271 (PMC6231669; doi:10.1371/journal.pone.0207271)
Supplement: S5 Table — (DOCX) [file pone.0207271.s005.docx]

S5 Table: Gene ontology analysis of the genes obtained within the SDR regions

| Gene ID | Gene Name | Items | Classification | Function | Description | Chromosome | Length (bp) |
| --- | --- | --- | --- | --- | --- | --- | --- |
| Gorai.001G088900 | CYP81D1 | GO:0055114 | Biological Process | oxidation-reduction process | Cytochrome P450 81D1 | Chr01 | 2,310 |
| Gorai.001G089000 | CYP81E8 | GO:0055114 | Biological Process | oxidation-reduction process | Cytochrome P450 81E8 | Chr01 | 2,160 |
| Gorai.001G121300 | PCMP-H60 | GO:0005488 | Molecular Function | binding | Pentatricopeptide repeat-containing protein At2g27610 | Chr01 | 3,092 |
| Gorai.001G121400 | SKD1 | GO:0017111 | Molecular Function | nucleoside-triphosphatase activity | Protein SUPPRESSOR OF K(+) TRANSPORT GROWTH DEFECT 1 | Chr01 | 5,167 |
| Gorai.001G121500 | SAP3 | GO:0008270 | Molecular Function | zinc ion binding | Zinc finger A20 and AN1 domain-containing stress-associated protein 3 | Chr01 | 1,413 |
| Gorai.001G121600 | GALT29A | GO:0030173 | Cellular Component | integral to Golgi membrane | Beta-1,6-galactosyltransferase GALT29A | Chr01 | 2,262 |
| Gorai.001G122200 | At1g67190 | GO:0005515 | Molecular Function | protein binding | F-box/LRR-repeat protein At1g67190 | Chr01 | 4,548 |
| Gorai.001G122300 | At2g27500 | GO:0043169 | Molecular Function | cation binding | Glucan endo-1,3-beta-glucosidase 14 | Chr01 | 3,164 |
| Gorai.002G229800 | CTPA3 | GO:0008236 | Molecular Function | serine-type peptidase activity | Carboxyl-terminal-processing peptidase 3, chloroplastic | Chr02 | 7,427 |
| Gorai.002G229900 | lst8 | GO:0005515 | Molecular Function | protein binding | Protein LST8 homolog | Chr02 | 5,370 |
| Gorai.002G231300 | SDC | GO:0030170 | Molecular Function | pyridoxal phosphate binding | Serine decarboxylase | Chr02 | 2,988 |
| Gorai.002G231500 | GT-2 | GO:0003677 | Molecular Function | DNA binding | Trihelix transcription factor GT-2 | Chr02 | 2,149 |
| Gorai.002G231600 | TKPR2 | GO:0050662 | Molecular Function | coenzyme binding | Tetraketide alpha-pyrone reductase 2 | Chr02 | 6,339 |
| Gorai.002G234400 | NFYA10 | GO:0006355 | Biological Process | regulation of transcription, DNA-dependent | Nuclear transcription factor Y subunit A-10 | Chr02 | 4,482 |
| Gorai.002G234500 | At1g74360 | GO:0005515 | Molecular Function | protein binding | Probable LRR receptor-like serine/threonine-protein kinase At1g74360 | Chr02 | 2,203 |
| Gorai.002G234600 | CAD1-A | GO:0016829 | Molecular Function | lyase activity | (+)-delta-cadinene synthase isozyme A | Chr02 | 12,460 |
| Gorai.002G235100 | DGD1 | GO:0009058 | Biological Process | biosynthetic process | Digalactosyldiacylglycerol synthase 1, chloroplastic | Chr02 | 7,151 |
| Gorai.002G235300 | TMK3 | GO:0016772 | Molecular Function | transferase activity, transferring phosphorus-containing groups | Receptor-like kinase TMK3 | Chr02 | 3,876 |
| Gorai.002G235500 | NUDT27 | GO:0016787 | Molecular Function | hydrolase activity | Nudix hydrolase 27, chloroplastic | Chr02 | 3,175 |
| Gorai.002G237800 | PRCP | GO:0008236 | Molecular Function | serine-type peptidase activity | Lysosomal Pro-X carboxypeptidase | Chr02 | 6,793 |
| Gorai.002G241100 | CYP94C1 | GO:0055114 | Biological Process | oxidation-reduction process | Cytochrome P450 94C1 | Chr02 | 1,735 |
| Gorai.002G241200 | RPP2B | GO:0006414 | Biological Process | translational elongation | 60S acidic ribosomal protein P2B | Chr02 | 1,809 |
| Gorai.002G241300 | AFRR | GO:0055114 | Biological Process | oxidation-reduction process | Monodehydroascorbate reductase | Chr02 | 3,324 |
| Gorai.002G241400 | SBEI | GO:0043169 | Molecular Function | cation binding | 1,4-alpha-glucan-branching enzyme 1, chloroplastic/amyloplastic | Chr02 | 10,835 |
| Gorai.003G137300 | ATX1 | GO:0046872 | Molecular Function | metal ion binding | Copper transport protein ATX1 | Chr03 | 1,085 |
| Gorai.003G137400 | RPL35 | GO:0006412 | Biological Process | translation | 60S ribosomal protein L35 | Chr03 | 1,507 |
| Gorai.003G137500 | EXO70A1 | GO:0006887 | Biological Process | exocytosis | Exocyst complex component EXO70A1 | Chr03 | 2,132 |
| Gorai.003G137600 | At5g02620 | GO:0005515 | Molecular Function | protein binding | Ankyrin repeat-containing protein At5g02620 | Chr03 | 1,841 |
| Gorai.006G021900 | BPA1 | GO:0003676 | Molecular Function | nucleic acid binding | Binding partner of ACD11 1 | Chr06 | 4,414 |
| Gorai.006G023400 | PPIP5K1 | GO:0046872 | Molecular Function | metal ion binding | Inositol hexakisphosphate and diphosphoinositol-pentakisphosphate kinase 1 | Chr06 | 13,117 |
| Gorai.006G024400 | At1g80170 | GO:0005975 | Biological Process | carbohydrate metabolic process | Probable polygalacturonase At1g80170 | Chr06 | 2,166 |
| Gorai.006G024600 | At5g15080 | GO:0016772 | Molecular Function | transferase activity, transferring phosphorus-containing groups | Probable receptor-like protein kinase At5g15080 | Chr06 | 4,563 |
| Gorai.006G024700 | NA | GO:0055085 | Biological Process | transmembrane transport | Mitochondrial outer membrane protein porin of 34 kDa | Chr06 | 2,746 |
| Gorai.006G032700 | RST1 | GO:0005488 | Molecular Function | binding | Protein RST1 | Chr06 | 15,077 |
| Gorai.006G032800 | At3g27700 | GO:0008270 | Molecular Function | zinc ion binding | Zinc finger CCCH domain-containing protein 41 | Chr06 | 8,553 |
| Gorai.006G032900 | RFS5 | GO:0003824 | Molecular Function | catalytic activity | Probable galactinol--sucrose galactosyltransferase 5 | Chr06 | 2,751 |
| Gorai.006G069600 | NA | GO:0006412 | Biological Process | translation | 40S ribosomal protein S3a | Chr06 | 3,366 |
| Gorai.006G081800 | NA | GO:0055114 | Biological Process | oxidation-reduction process | Cytochrome P450 CYP736A12 | Chr06 | 1,542 |
| Gorai.006G084300 | CRK26 | GO:0016772 | Molecular Function | transferase activity, transferring phosphorus-containing groups | Cysteine-rich receptor-like protein kinase 26 | Chr06 | 3,165 |
| Gorai.006G099500 | Ank2 | GO:0005515 | Molecular Function | protein binding | Ankyrin-2 | Chr06 | 2,655 |
| Gorai.006G099700 | AGD14 | GO:0032312 | Biological Process | regulation of ARFGTPase activity | Probable ADP-ribosylation factor GTPase-activating protein AGD14 | Chr06 | 868 |
| Gorai.007G221600 | At5g67385 | GO:0009416 | Biological Process | response to light stimulus | BTB/POZ domain-containing protein At5g67385 | Chr07 | 3,289 |
| Gorai.007G221800 | NA | GO:0070461 | Cellular Component | SAGA-type complex | NA | Chr07 | 2,127 |
| Gorai.007G346300 | WOX11 | GO:0043565 | Molecular Function | sequence-specific DNA binding | WUSCHEL-related homeobox 11 | Chr07 | 2,252 |
| Gorai.007G346400 | MYB44 | GO:0005515 | Molecular Function | protein binding | Transcription factor MYB44 | Chr07 | 1,871 |
| Gorai.007G347100 | DAPB2 | GO:0070402 | Molecular Function | NADPH binding | 4-hydroxy-tetrahydrodipicolinate reductase 2, chloroplastic | Chr07 | 5,757 |
| Gorai.007G347200 | LHP1 | GO:0005634 | Cellular Component | nucleus | Chromo domain-containing protein LHP1 | Chr07 | 3,457 |
| Gorai.007G347300 | SIGB | GO:0016987 | Molecular Function | sigma factor activity | RNA polymerase sigma factor sigB | Chr07 | 4,086 |
| Gorai.007G347600 | TFCA | GO:0051082 | Molecular Function | unfolded protein binding | Tubulin-folding cofactor A | Chr07 | 2,050 |
| Gorai.007G347700 | CYP89A2 | GO:0055114 | Biological Process | oxidation-reduction process | Cytochrome P450 89A2 | Chr07 | 1,542 |
| Gorai.007G347800 | CYP89A2 | GO:0055114 | Biological Process | oxidation-reduction process | Cytochrome P450 89A2 | Chr07 | 1,815 |
| Gorai.007G348600 | MYB39 | GO:0005515 | Molecular Function | protein binding | Transcription factor MYB39 | Chr07 | 1,654 |
| Gorai.007G348800 | NA | GO:0016772 | Molecular Function | transferase activity, transferring phosphorus-containing groups | NA | Chr07 | 6,112 |
| Gorai.007G349000 | NA | GO:0007264 | Biological Process | small GTPase mediated signal transduction | ADP-ribosylation factor | Chr07 | 6,440 |
| Gorai.007G349200 | BMY1 | GO:0043169 | Molecular Function | cation binding | Beta-amylase | Chr07 | 4,844 |
| Gorai.007G349400 | At3g07070 | GO:0016772 | Molecular Function | transferase activity, transferring phosphorus-containing groups | Serine/threonine-protein kinase At3g07070 | Chr07 | 3,947 |
| Gorai.007G350700 | ARI7 | GO:0008270 | Molecular Function | zinc ion binding | Probable E3 ubiquitin-protein ligase ARI7 | Chr07 | 8,960 |
| Gorai.007G350800 | UBC22 | GO:0016881 | Molecular Function | acid-amino acid ligase activity | Ubiquitin-conjugating enzyme E2 22 | Chr07 | 4,371 |
| Gorai.007G350900 | SUVH1 | GO:0042393 | Molecular Function | Histone binding | Histone-lysine N-methyltransferase, H3 lysine-9 specific SUVH1 | Chr07 | 4,706 |
| Gorai.007G353100 | CENPE | GO:0007018 | Biological Process | microtubule-based movement | Centromere-associated protein E | Chr07 | 20,870 |
| Gorai.007G353200 | Nanp | GO:0016787 | Molecular Function | Hydrolase activity | N-acylneuraminate-9-phosphatase | Chr07 | 4,324 |
| Gorai.007G353300 | nol10 | GO:0005634 | Cellular Component | nucleus | Nucleolar protein 10 | Chr07 | 7,970 |
| Gorai.007G356000 | At4g27220 | GO:0043531 | Molecular Function | ADP binding | Probable disease resistance protein At4g27220 | Chr07 | 17,213 |
| Gorai.009G366600 | NA | GO:0005515 | Molecular Function | protein binding | NA | Chr09 | 3,414 |
| Gorai.009G367200 | TULP7 | GO:0005515 | Molecular Function | protein binding | Tubby-like F-box protein 7 | Chr09 | 3,662 |
| Gorai.009G367300 | MBF1B | GO:0043565 | Molecular Function | sequence-specific DNA binding | Multiprotein-bridging factor 1b | Chr09 | 2,290 |
| Gorai.009G374600 | RABA1F | GO:0016020 | Cellular Component | membrane | Ras-related protein RABA1f | Chr09 | 2,890 |
| Gorai.010G007500 | CKX5 | GO:0055114 | Biological Process | oxidation-reduction process | Cytokinin dehydrogenase 5 | Chr10 | 4,568 |
| Gorai.010G007600 | rsmI | GO:0008168 | Molecular Function | Methyl transferase activity | Ribosomal RNA small subunit methyltransferase I | Chr10 | 4,272 |
| Gorai.010G007700 | BLH11 | GO:0043565 | Molecular Function | sequence-specific DNA binding | BEL1-like homeodomain protein 11 | Chr10 | 3,417 |
| Gorai.010G008100 | HHT1 | GO:0016747 | Molecular Function | transferaseactivity,transferringacylgroupsotherthanamino-acylgroups | Omega-hydroxypalmitate O-feruloyl transferase | Chr10 | 2,508 |
| Gorai.010G009700 | At1g19525 | GO:0005488 | Molecular Function | binding | Pentatricopeptide repeat-containing protein At1g19525 | Chr10 | 3,861 |
| Gorai.010G009900 | At1g75220 | GO:0055085 | Biological Process | Transmembrane transport | Sugar transporter ERD6-like 6 | Chr10 | 6,148 |
| Gorai.010G010000 | RL6 | GO:0005515 | Molecular Function | Protein binding | Protein RADIALIS-like 6 | Chr10 | 1,578 |
| Gorai.010G010100 | At2g16250 | GO:0016772 | Molecular Function | Transferase activity, transferring phosphorus-containing groups | Probable LRR receptor-like serine/threonine-protein kinase At2g16250 | Chr10 | 5,447 |
| Gorai.010G012000 | NAC053 | GO:0006355 | Biological Process | Regulation of transcription, DNA-dependent | NAC domain-containing protein 53 | Chr10 | 4,345 |
| Gorai.010G012200 | GA17800 | GO:0016020 | Cellular Component | membrane | Leishmanolysin-like peptidase | Chr10 | 6,386 |
| Gorai.011G135200 | NA | GO:0055114 | Biological Process | oxidation-reduction process | Stearoyl-[acyl-carrier-protein] 9-desaturase, chloroplastic | Chr11 | 5,729 |
| Gorai.011G135300 | IQD14 | GO:0005515 | Molecular Function | Protein binding | Protein IQ-DOMAIN 14 | Chr11 | 5,447 |
| Gorai.011G136900 | At3g11710 | GO:0006430 | Biological Process | lysyl-tRNA amino acylation | Lysine--tRNA ligase, cytoplasmic | Chr11 | 6,200 |
| Gorai.011G137100 | At1g68650 | GO:0016020 | Cellular Component | membrane | GDT1-like protein 5 | Chr11 | 374 |
| Gorai.011G137400 | TAF1 | GO:0005515 | Molecular Function | Protein binding | Transcription initiation factor TFIID subunit 1 | Chr11 | 18,721 |
| Gorai.011G137500 | EP3 | GO:0016998 | Biological Process | Cell wall macromolecule catabolic process | Endochitinase EP3 | Chr11 | 1,094 |
| Gorai.011G141100 | NA | GO:0050660 | Molecular Function | Flavin adenine dinucleotide binding | Acetolactate synthase 3, chloroplastic | Chr11 | 2,305 |
| Gorai.011G141200 | ycf2-A | GO:0009507 | Cellular Component | chloroplast | Protein Ycf2 | Chr11 | 1,383 |
| Gorai.011G142500 | ACR4 | GO:0016772 | Molecular Function | Transferase activity, transferring phosphorus-containing groups | Serine/threonine-protein kinase-like protein ACR4 | Chr11 | 3,635 |
| Gorai.011G142600 | GCN2 | GO:0016772 | Molecular Function | Transferase activity, transferring phosphorus-containing groups | Probable serine/threonine-protein kinase GCN2 | Chr11 | 17,400 |
| Gorai.011G142700 | NA | GO:0003676 | Molecular Function | Nucleic acid binding | NA | Chr11 | 3,096 |
| Gorai.011G154700 | PHT1-5 | GO:0055085 | Biological Process | Transmembrane transport | Probable inorganic phosphate transporter 1-5 | Chr11 | 1,771 |
| Gorai.011G158300 | LECRKS2 | GO:0016772 | Molecular Function | Transferase activity, transferring phosphorus-containing groups | Receptor like protein kinase S.2 | Chr11 | 2,499 |
| Gorai.011G158400 | CML22 | GO:0005509 | Molecular Function | Calcium ion binding | Probable calcium-binding protein CML22 | Chr11 | 2,588 |
| Gorai.011G160100 | NA | GO:0051082 | Molecular Function | Unfolded protein binding | NA | Chr11 | 3,056 |
| Gorai.011G162100 | RCH2 | GO:0016772 | Molecular Function | Transferase activity, transferring phosphorus-containing groups | Receptor-like protein kinase 2 | Chr11 | 4,066 |
| Gorai.011G162200 | BAK1 | GO:0016772 | Molecular Function | Transferase activity, transferring phosphorus-containing groups | BRASSINOSTEROID INSENSITIVE 1-associated receptor kinase 1 | Chr11 | 2,378 |
| Gorai.011G168600 | CLC-D | GO:0055085 | Biological Process | Transmembrane transport | Chloride channel protein CLC-d | Chr11 | 12,240 |
| Gorai.011G181800 | PPD | GO:0050242 | Molecular Function | pyruvate, phosphate dikinase activity | Pyruvate, phosphate dikinase, chloroplastic | Chr11 | 7,840 |
| Gorai.012G141400 | psaA | GO:0016021 | Cellular Component | Integral to membrane | Photosystem I P700 chlorophyll a apoprotein A1 | Chr12 | 459 |
| Gorai.012G141500 | poxN1 | GO:0055114 | Biological Process | oxidation-reduction process | Peroxidase N1 | Chr12 | 1,366 |
| Gorai.012G141600 | FOLB1 | GO:0006760 | Biological Process | Folic acid-containing compound metabolic process | Dihydroneopterin aldolase 1 | Chr12 | 2,078 |
