# Supplementary material for: SSR-Linkage map of interspecific populations derived from Gossypium trilobum and Gossypium thurberi and determination of genes harbored within the segregating distortion regions
Source: PLoS One. 2018 Nov 12;13(11):e0207271. doi: 10.1371/journal.pone.0207271 (PMC6231669; doi:10.1371/journal.pone.0207271)
Supplement: S6 Table — (DOCX) [file pone.0207271.s006.docx]

**Supplementary Table 1: Markers in inconformity between the genetic map and the physical map of *G. hirsutum* (GhDt)**

|  |  |  |  |  |  |
| --- | --- | --- | --- | --- | --- |
|  | **Genetic location (cM)** | | **Physical location (Bp)** | |  |
| Markers | **Linkage group** | **Location** | **Chromosome** | **Start** | **End** |
| SWU11099 | G_Chr02 | 0 | P_01 | 8108527 | 8108695 |
| SWU11160 | G_Chr02 | 5.464 | P_01 | 11021285 | 11021457 |
| SWU11559 | G_Chr02 | 6.558 | P_01 | 48793205 | 48793406 |
| SWU11492 | G_Chr02 | 7.111 | P_01 | 42691875 | 42691977 |
| SWU11319 | G_Chr02 | 7.17 | P_01 | 22968722 | 22968915 |
| SWU11342 | G_Chr02 | 7.173 | P_01 | 26107087 | 26107224 |
| SWU11279 | G_Chr02 | 7.348 | P_01 | 18945200 | 18945373 |
| SWU11210 | G_Chr02 | 8.012 | P_01 | 13813874 | 13814062 |
| SWU12229 | G_Chr02 | 8.746 | P_01 | 49013255 | 49013437 |
| SWU11614 | G_Chr02 | 9.652 | P_01 | 53557915 | 53558103 |
| SWU21545 | G_Chr02 | 19.215 | P_01 | 57643260 | 57643495 |
| SWU11708 | G_Chr02 | 23.968 | P_01 | 58086192 | 58086344 |
| SWU11710 | G_Chr02 | 24.436 | P_01 | 58175027 | 58175179 |
| SWU11711 | G_Chr02 | 25.127 | P_01 | 58237232 | 58237391 |
| SWU11720 | G_Chr02 | 26.049 | P_01 | 58671864 | 58672029 |
| SWU11734 | G_Chr02 | 28.665 | P_01 | 59079349 | 59079509 |
| SWU12229 | G_Chr03 | 32.304 | P_01 | 49013255 | 49013437 |
| SWU10330 | G_Chr08 | 36.173 | P_01 | 24662881 | 24662947 |
| SWU10330 | G_Chr08 | 36.173 | P_01 | 7578809 | 7578869 |
| SWU16992 | G_Chr08 | 66.209 | P_01 | 3336684 | 3336782 |
| SWU14306 | G_Chr03 | 32.028 | P_02 | 39233002 | 39233134 |
| SWU13644 | G_Chr04 | 7.975 | P_02 | 6620811 | 6620936 |
| SWU13921 | G_Chr05 | 0 | P_02 | 1769781 | 1769920 |
| SWU13939 | G_Chr05 | 3.806 | P_02 | 2820399 | 2820592 |
| SWU13939 | G_Chr05 | 3.806 | P_02 | 2812971 | 2813161 |
| SWU13942 | G_Chr05 | 4.897 | P_02 | 2890830 | 2890952 |
| SWU13947 | G_Chr05 | 5.632 | P_02 | 3076552 | 3076715 |
| SWU13993 | G_Chr05 | 22.71 | P_02 | 5306271 | 5306436 |
| SWU13993 | G_Chr05 | 22.71 | P_02 | 5301617 | 5301791 |
| SWU13993 | G_Chr05 | 22.71 | P_02 | 5296431 | 5296598 |
| SWU13990 | G_Chr05 | 23.206 | P_02 | 5172911 | 5173118 |
| SWU14003 | G_Chr05 | 24.421 | P_02 | 5791488 | 5791613 |
| SWU14306 | G_Chr05 | 27.127 | P_02 | 39233002 | 39233134 |
| SWU14016 | G_Chr05 | 28.873 | P_02 | 6598954 | 6599128 |
| SWU14013 | G_Chr05 | 29.63 | P_02 | 6397212 | 6397410 |
| SWU14017 | G_Chr05 | 30.102 | P_02 | 6655076 | 6655243 |
| SWU14046 | G_Chr05 | 33.526 | P_02 | 9047342 | 9047538 |
| SWU14042 | G_Chr05 | 33.814 | P_02 | 8570264 | 8570437 |
| SWU14035 | G_Chr05 | 34.158 | P_02 | 8344806 | 8344995 |
| SWU14261 | G_Chr05 | 35.209 | P_02 | 28967998 | 28968171 |
| SWU14054 | G_Chr05 | 36.113 | P_02 | 9929597 | 9929747 |
| SWU14428 | G_Chr05 | 36.94 | P_02 | 46581534 | 46581718 |
| SWU14087 | G_Chr05 | 37.334 | P_02 | 12083515 | 12083679 |
| SWU14079 | G_Chr05 | 37.412 | P_02 | 11343242 | 11343395 |
| SWU14091 | G_Chr05 | 37.699 | P_02 | 12418694 | 12418800 |
| SWU14093 | G_Chr05 | 37.699 | P_02 | 12625370 | 12625556 |
| SWU14098 | G_Chr05 | 37.699 | P_02 | 13000885 | 13001020 |
| SWU14100 | G_Chr05 | 37.699 | P_02 | 13097549 | 13097663 |
| SWU14140 | G_Chr05 | 37.945 | P_02 | 16101438 | 16101594 |
| SWU14141 | G_Chr05 | 37.955 | P_02 | 16106345 | 16106481 |
| SWU14142 | G_Chr05 | 37.961 | P_02 | 16137277 | 16137456 |
| SWU14194 | G_Chr05 | 38.227 | P_02 | 21866464 | 21866613 |
| SWU14225 | G_Chr05 | 38.366 | P_02 | 25021256 | 25021414 |
| SWU14206 | G_Chr05 | 38.506 | P_02 | 23513383 | 23513502 |
| SWU14208 | G_Chr05 | 38.523 | P_02 | 23615585 | 23615852 |
| SWU14208 | G_Chr05 | 38.523 | P_02 | 23615585 | 23615774 |
| SWU14230 | G_Chr05 | 38.566 | P_02 | 25620457 | 25620650 |
| SWU14220 | G_Chr05 | 38.568 | P_02 | 24784603 | 24784763 |
| SWU14363 | G_Chr05 | 38.678 | P_02 | 39852532 | 39852709 |
| SWU14377 | G_Chr05 | 38.74 | P_02 | 41222597 | 41222762 |
| SWU14370 | G_Chr05 | 38.807 | P_02 | 40173902 | 40174026 |
| SWU14333 | G_Chr05 | 38.818 | P_02 | 36830202 | 36830306 |
| SWU14365 | G_Chr05 | 38.818 | P_02 | 39977543 | 39977664 |
| SWU14352 | G_Chr05 | 38.844 | P_02 | 34602122 | 34602309 |
| SWU14305 | G_Chr05 | 38.895 | P_02 | 39309075 | 39309196 |
| SWU14222 | G_Chr05 | 38.954 | P_02 | 24813299 | 24813440 |
| SWU14397 | G_Chr05 | 39.19 | P_02 | 43807387 | 43807556 |
| SWU14404 | G_Chr05 | 39.318 | P_02 | 44560694 | 44560847 |
| SWU14447 | G_Chr05 | 39.957 | P_02 | 47326561 | 47326730 |
| SWU14466 | G_Chr05 | 40.128 | P_02 | 50487699 | 50487897 |
| SWU14496 | G_Chr05 | 40.423 | P_02 | 52807465 | 52807620 |
| SWU14500 | G_Chr05 | 40.613 | P_02 | 53154588 | 53154766 |
| SWU14526 | G_Chr05 | 40.808 | P_02 | 55538445 | 55538599 |
| SWU14523 | G_Chr05 | 40.921 | P_02 | 55311776 | 55311923 |
| SWU14488 | G_Chr05 | 41.229 | P_02 | 52242488 | 52242658 |
| SWU14481 | G_Chr05 | 41.701 | P_02 | 51797722 | 51797855 |
| SWU14540 | G_Chr05 | 42.165 | P_02 | 56594474 | 56594672 |
| SWU14551 | G_Chr05 | 42.708 | P_02 | 57343977 | 57344164 |
| SWU14548 | G_Chr05 | 42.891 | P_02 | 57141230 | 57141414 |
| SWU14566 | G_Chr05 | 43.171 | P_02 | 58086936 | 58087117 |
| SWU14588 | G_Chr05 | 43.659 | P_02 | 59901567 | 59901727 |
| SWU14597 | G_Chr05 | 44.012 | P_02 | 60478207 | 60478379 |
| SWU14582 | G_Chr05 | 44.135 | P_02 | 59601115 | 59601290 |
| SWU14625 | G_Chr05 | 44.552 | P_02 | 62300078 | 62300210 |
| SWU14601 | G_Chr05 | 44.76 | P_02 | 60916655 | 60916833 |
| SWU14629 | G_Chr05 | 44.978 | P_02 | 62634606 | 62634750 |
| SWU14638 | G_Chr05 | 46.828 | P_02 | 63448022 | 63448185 |
| SWU14663 | G_Chr05 | 50.715 | P_02 | 64382548 | 64382628 |
| SWU14665 | G_Chr05 | 51.166 | P_02 | 64455516 | 64455675 |
| SWU14679 | G_Chr05 | 58.806 | P_02 | 64681371 | 64681525 |
| SWU14686 | G_Chr05 | 64.25 | P_02 | 64891990 | 64892177 |
| SWU14692 | G_Chr05 | 68.391 | P_02 | 65150408 | 65150583 |
| SWU14698 | G_Chr05 | 71.594 | P_02 | 65409262 | 65409407 |
| SWU14706 | G_Chr05 | 74.739 | P_02 | 65686739 | 65686912 |
| SWU14710 | G_Chr05 | 75.789 | P_02 | 65737449 | 65737581 |
| SWU14715 | G_Chr05 | 79.957 | P_02 | 65909897 | 65910073 |
| SWU14737 | G_Chr05 | 88.077 | P_02 | 66785375 | 66785541 |
| SWU14744 | G_Chr05 | 92.563 | P_02 | 67052912 | 67053123 |
| SWU14987 | G_Chr06 | 0 | P_02 | 55794508 | 55794691 |
| SWU14987 | G_Chr06 | 9.117 | P_02 | 55794508 | 55794691 |
| SWU15193 | G_Chr07 | 28.58 | P_02 | 55490932 | 55491104 |
| SWU15193 | G_Chr07 | 28.58 | P_02 | 55483043 | 55483213 |
| SWU14289 | G_Chr07 | 33.571 | P_02 | 48488082 | 48488274 |
| SWU14046 | G_Chr11 | 42.846 | P_02 | 9047342 | 9047538 |
| SWU21785 | G_Chr13 | 67.639 | P_02 | 65706064 | 65706188 |
| SWU12229 | G_Chr02 | 8.746 | P_03 | 12243632 | 12243818 |
| SWU12135 | G_Chr06 | 0 | P_03 | 7685538 | 7685711 |
| SWU14989 | G_Chr06 | 9.426 | P_03 | 12528583 | 12528716 |
| SWU22383 | G_Chr13 | 45.492 | P_03 | 9912507 | 9912678 |
| SWU12058 | G_Chr13 | 49.895 | P_03 | 4706766 | 4706923 |
| SWU12482 | G_Chr13 | 55.598 | P_03 | 16558592 | 16558793 |
| SWU14306 | G_Chr03 | 32.028 | P_04 | 42359156 | 42359279 |
| SWU14306 | G_Chr05 | 27.127 | P_04 | 42359156 | 42359279 |
| SWU15131 | G_Chr06 | 2.6 | P_04 | 26249516 | 26249636 |
| SWU16010 | G_Chr07 | 25.915 | P_04 | 1866141 | 1866341 |
| SWU18710 | G_Chr09 | 0.28 | P_04 | 25288152 | 25288320 |
| SWU18710 | G_Chr09 | 0.28 | P_04 | 25288152 | 25288273 |
| SWU18750 | G_Chr09 | 0.28 | P_04 | 28646822 | 28646949 |
| SWU18726 | G_Chr09 | 0.333 | P_04 | 26351501 | 26351683 |
| SWU18760 | G_Chr09 | 1.064 | P_04 | 29663195 | 29663380 |
| SWU15131 | G_Chr10 | 38.761 | P_04 | 26249516 | 26249636 |
| SWU20407 | G_Chr11 | 52.407 | P_04 | 29710869 | 29711030 |
| SWU20999 | G_Chr12 | 0 | P_04 | 8391083 | 8391263 |
| SWU20984 | G_Chr12 | 3.336 | P_04 | 7726142 | 7726321 |
| SWU20881 | G_Chr12 | 6.232 | P_04 | 3232908 | 3233095 |
| SWU20881 | G_Chr12 | 6.232 | P_04 | 3230762 | 3230949 |
| SWU20976 | G_Chr12 | 6.438 | P_04 | 7428554 | 7428721 |
| SWU20880 | G_Chr12 | 6.703 | P_04 | 3252025 | 3252213 |
| SWU20756 | G_Chr12 | 15.207 | P_04 | 220532 | 220712 |
| SWU20774 | G_Chr12 | 15.274 | P_04 | 956687 | 956839 |
| SWU20752 | G_Chr12 | 15.815 | P_04 | 164461 | 164629 |
| SWU21669 | G_Chr12 | 19.002 | P_04 | 50464203 | 50464390 |
| SWU21646 | G_Chr12 | 35.952 | P_04 | 50100991 | 50101175 |
| SWU21626 | G_Chr12 | 38.62 | P_04 | 49294558 | 49294693 |
| SWU21616 | G_Chr12 | 40.817 | P_04 | 49052714 | 49052869 |
| SWU21618 | G_Chr12 | 40.932 | P_04 | 49077779 | 49077905 |
| SWU21608 | G_Chr12 | 41.213 | P_04 | 48915010 | 48915209 |
| SWU21584 | G_Chr12 | 42.736 | P_04 | 48131104 | 48131270 |
| SWU21584 | G_Chr12 | 42.736 | P_04 | 48124951 | 48125126 |
| SWU21581 | G_Chr12 | 44.681 | P_04 | 48091951 | 48092087 |
| SWU21574 | G_Chr12 | 44.933 | P_04 | 47952855 | 47953023 |
| SWU21511 | G_Chr12 | 45.083 | P_04 | 46250755 | 46250848 |
| SWU21402 | G_Chr12 | 47.974 | P_04 | 42566135 | 42566333 |
| SWU21379 | G_Chr12 | 49.438 | P_04 | 41492572 | 41492773 |
| SWU21374 | G_Chr12 | 49.986 | P_04 | 41236104 | 41236260 |
| SWU21331 | G_Chr12 | 50.57 | P_04 | 38761673 | 38761772 |
| SWU21354 | G_Chr12 | 50.57 | P_04 | 39850760 | 39850940 |
| SWU21225 | G_Chr12 | 50.763 | P_04 | 17486300 | 17486454 |
| SWU21351 | G_Chr12 | 51.196 | P_04 | 39764701 | 39764900 |
| SWU21180 | G_Chr12 | 65.054 | P_04 | 15453843 | 15454035 |
| SWU21178 | G_Chr12 | 65.267 | P_04 | 15419001 | 15419172 |
| SWU21173 | G_Chr12 | 70.914 | P_04 | 15158341 | 15158530 |
| SWU21072 | G_Chr12 | 79.718 | P_04 | 10932092 | 10932266 |
| SWU21004 | G_Chr12 | 88.288 | P_04 | 8491998 | 8492163 |
| SWU16010 | G_Chr13 | 19.276 | P_04 | 1866141 | 1866341 |
| SWU21785 | G_Chr13 | 67.639 | P_04 | 48653895 | 48654028 |
| SWU10330 | G_Chr01 | 51.517 | P_05 | 46851491 | 46851551 |
| SWU18284 | G_Chr01 | 52.019 | P_05 | 24243592 | 24243761 |
| SWU18938 | G_Chr04 | 0 | P_05 | 61431333 | 61431493 |
| SWU18954 | G_Chr04 | 3.422 | P_05 | 61694447 | 61694536 |
| SWU16010 | G_Chr07 | 25.915 | P_05 | 48410333 | 48410533 |
| SWU16010 | G_Chr07 | 25.915 | P_05 | 8413979 | 8414179 |
| SWU10330 | G_Chr08 | 36.173 | P_05 | 46851491 | 46851551 |
| SWU17655 | G_Chr09 | 0 | P_05 | 609977 | 610119 |
| SWU18750 | G_Chr09 | 0.28 | P_05 | 34143922 | 34144052 |
| SWU17677 | G_Chr09 | 4.063 | P_05 | 1198587 | 1198754 |
| SWU17682 | G_Chr09 | 5.699 | P_05 | 1475214 | 1475327 |
| SWU17721 | G_Chr09 | 7.116 | P_05 | 2653417 | 2653570 |
| SWU17705 | G_Chr09 | 8.61 | P_05 | 2287168 | 2287314 |
| SWU17707 | G_Chr09 | 8.69 | P_05 | 2305389 | 2305568 |
| SWU17714 | G_Chr09 | 8.69 | P_05 | 2444562 | 2444753 |
| SWU17742 | G_Chr09 | 10.423 | P_05 | 3162490 | 3162621 |
| SWU17750 | G_Chr09 | 11.316 | P_05 | 3457341 | 3457518 |
| SWU17790 | G_Chr09 | 16.706 | P_05 | 5077762 | 5077920 |
| SWU17793 | G_Chr09 | 17.469 | P_05 | 5192076 | 5192239 |
| SWU17799 | G_Chr09 | 18.525 | P_05 | 5350838 | 5350999 |
| SWU17801 | G_Chr09 | 18.525 | P_05 | 5351446 | 5351582 |
| SWU17816 | G_Chr09 | 19.412 | P_05 | 6137053 | 6137242 |
| SWU17827 | G_Chr09 | 21.108 | P_05 | 6540922 | 6541095 |
| SWU17848 | G_Chr09 | 23.089 | P_05 | 7086589 | 7086761 |
| SWU17895 | G_Chr09 | 29.51 | P_05 | 8511433 | 8511598 |
| SWU17924 | G_Chr09 | 30.993 | P_05 | 9677477 | 9677667 |
| SWU17935 | G_Chr09 | 32.42 | P_05 | 10140650 | 10140811 |
| SWU17937 | G_Chr09 | 33.055 | P_05 | 10302663 | 10302804 |
| SWU17938 | G_Chr09 | 33.08 | P_05 | 10309466 | 10309643 |
| SWU17947 | G_Chr09 | 33.465 | P_05 | 10511358 | 10511518 |
| SWU17949 | G_Chr09 | 34.161 | P_05 | 10635501 | 10635687 |
| SWU17955 | G_Chr09 | 35.151 | P_05 | 10817494 | 10817639 |
| SWU17981 | G_Chr09 | 38.346 | P_05 | 11704471 | 11704570 |
| SWU17948 | G_Chr09 | 38.582 | P_05 | 10626568 | 10626751 |
| SWU17984 | G_Chr09 | 39.637 | P_05 | 11826587 | 11826737 |
| SWU18019 | G_Chr09 | 42.65 | P_05 | 12789515 | 12789639 |
| SWU18025 | G_Chr09 | 42.826 | P_05 | 12890665 | 12890851 |
| SWU18026 | G_Chr09 | 43.655 | P_05 | 12929048 | 12929237 |
| SWU18027 | G_Chr09 | 43.655 | P_05 | 12971433 | 12971608 |
| SWU18027 | G_Chr09 | 43.655 | P_05 | 12954877 | 12955054 |
| SWU18039 | G_Chr09 | 43.655 | P_05 | 13339793 | 13339991 |
| SWU18103 | G_Chr09 | 45.843 | P_05 | 15929730 | 15929898 |
| SWU18127 | G_Chr09 | 46.778 | P_05 | 16822095 | 16822286 |
| SWU18160 | G_Chr09 | 52.918 | P_05 | 18225950 | 18226040 |
| SWU18178 | G_Chr09 | 53.84 | P_05 | 19186133 | 19186279 |
| SWU18253 | G_Chr09 | 67.129 | P_05 | 22359029 | 22359219 |
| SWU18288 | G_Chr09 | 68.512 | P_05 | 24392123 | 24392288 |
| SWU18265 | G_Chr09 | 70.501 | P_05 | 23251765 | 23251963 |
| SWU18298 | G_Chr09 | 72.077 | P_05 | 24716665 | 24716859 |
| SWU18314 | G_Chr09 | 75.111 | P_05 | 25521408 | 25521579 |
| SWU18323 | G_Chr09 | 76.328 | P_05 | 26021083 | 26021191 |
| SWU18335 | G_Chr09 | 79.231 | P_05 | 26965057 | 26965229 |
| SWU18350 | G_Chr09 | 79.735 | P_05 | 27388983 | 27389131 |
| SWU18389 | G_Chr09 | 82.782 | P_05 | 29682312 | 29682517 |
| SWU18424 | G_Chr09 | 83.355 | P_05 | 31570224 | 31570394 |
| SWU18626 | G_Chr09 | 85.84 | P_05 | 51600422 | 51600601 |
| SWU18453 | G_Chr09 | 85.984 | P_05 | 33281212 | 33281343 |
| SWU18499 | G_Chr09 | 86.414 | P_05 | 36877038 | 36877218 |
| SWU18603 | G_Chr09 | 86.688 | P_05 | 50140727 | 50140906 |
| SWU18598 | G_Chr09 | 86.717 | P_05 | 49555109 | 49555280 |
| SWU18598 | G_Chr09 | 86.717 | P_05 | 49555109 | 49555227 |
| SWU18593 | G_Chr09 | 86.765 | P_05 | 47995357 | 47995538 |
| SWU18545 | G_Chr09 | 86.806 | P_05 | 43028398 | 43028539 |
| SWU18558 | G_Chr09 | 86.856 | P_05 | 44441425 | 44441615 |
| SWU18568 | G_Chr09 | 86.903 | P_05 | 45247987 | 45248135 |
| SWU18502 | G_Chr09 | 86.975 | P_05 | 37262978 | 37263117 |
| SWU18584 | G_Chr09 | 87.006 | P_05 | 47008366 | 47008503 |
| SWU18507 | G_Chr09 | 87.025 | P_05 | 37830068 | 37830239 |
| SWU18528 | G_Chr09 | 87.121 | P_05 | 41269168 | 41269279 |
| SWU18494 | G_Chr09 | 87.139 | P_05 | 36553103 | 36553273 |
| SWU18489 | G_Chr09 | 87.194 | P_05 | 36219264 | 36219428 |
| SWU18493 | G_Chr09 | 87.198 | P_05 | 36457531 | 36457685 |
| SWU18437 | G_Chr09 | 87.856 | P_05 | 32445063 | 32445248 |
| SWU18439 | G_Chr09 | 87.888 | P_05 | 32465342 | 32465505 |
| SWU18616 | G_Chr09 | 88.314 | P_05 | 51006271 | 51006452 |
| SWU18638 | G_Chr09 | 89.406 | P_05 | 52419024 | 52419121 |
| SWU18576 | G_Chr09 | 89.668 | P_05 | 46037352 | 46037528 |
| SWU18659 | G_Chr09 | 92.126 | P_05 | 53866911 | 53867136 |
| SWU18659 | G_Chr09 | 92.126 | P_05 | 54111118 | 54111289 |
| SWU18640 | G_Chr09 | 92.428 | P_05 | 52546923 | 52547111 |
| SWU18622 | G_Chr09 | 93.909 | P_05 | 51316907 | 51317057 |
| SWU18622 | G_Chr09 | 93.909 | P_05 | 51415872 | 51416029 |
| SWU18671 | G_Chr09 | 96.559 | P_05 | 55384072 | 55384190 |
| SWU18671 | G_Chr09 | 96.559 | P_05 | 54561834 | 54561973 |
| SWU16010 | G_Chr13 | 19.276 | P_05 | 48410333 | 48410533 |
| SWU16010 | G_Chr13 | 47.694 | P_05 | 8413979 | 8414179 |
| SWU22383 | G_Chr13 | 45.492 | P_05 | 58427377 | 58427548 |
| SWU16010 | G_Chr13 | 19.276 | P_05 | 48410333 | 48410533 |
| SWU16010 | G_Chr13 | 47.694 | P_05 | 8413979 | 8414179 |
| SWU14194 | G_Chr05 | 38.227 | P_06 | 53819322 | 53819455 |
| SWU18750 | G_Chr09 | 0.28 | P_06 | 9438318 | 9438448 |
| SWU18974 | G_Chr10 | 0 | P_06 | 270859 | 270989 |
| SWU18979 | G_Chr10 | 3.353 | P_06 | 549216 | 549409 |
| SWU18986 | G_Chr10 | 5.571 | P_06 | 831139 | 831256 |
| SWU19014 | G_Chr10 | 12.2 | P_06 | 1566125 | 1566300 |
| SWU19013 | G_Chr10 | 12.632 | P_06 | 1520091 | 1520268 |
| SWU19008 | G_Chr10 | 13.737 | P_06 | 1381090 | 1381282 |
| SWU19030 | G_Chr10 | 21.753 | P_06 | 2113056 | 2113209 |
| SWU19040 | G_Chr10 | 25.306 | P_06 | 2343851 | 2344030 |
| SWU19058 | G_Chr10 | 29.546 | P_06 | 2741170 | 2741364 |
| SWU19073 | G_Chr10 | 36.498 | P_06 | 3277058 | 3277233 |
| SWU19087 | G_Chr10 | 38.212 | P_06 | 4406990 | 4407163 |
| SWU15131 | G_Chr10 | 38.761 | P_06 | 37589108 | 37589236 |
| SWU15131 | G_Chr10 | 38.761 | P_06 | 37573808 | 37573938 |
| SWU19110 | G_Chr10 | 40.056 | P_06 | 5216344 | 5216528 |
| SWU19113 | G_Chr10 | 40.787 | P_06 | 5282796 | 5282960 |
| SWU19125 | G_Chr10 | 42.561 | P_06 | 5745422 | 5745530 |
| SWU19128 | G_Chr10 | 43.619 | P_06 | 5848273 | 5848456 |
| SWU19144 | G_Chr10 | 44.872 | P_06 | 6525331 | 6525528 |
| SWU19148 | G_Chr10 | 45.809 | P_06 | 6843616 | 6843800 |
| SWU19185 | G_Chr10 | 47.911 | P_06 | 9192387 | 9192555 |
| SWU19610 | G_Chr10 | 49.24 | P_06 | 50866030 | 50866212 |
| SWU19596 | G_Chr10 | 50.382 | P_06 | 48812104 | 48812235 |
| SWU19665 | G_Chr10 | 50.701 | P_06 | 55508397 | 55508594 |
| SWU19588 | G_Chr10 | 51.352 | P_06 | 47436501 | 47436650 |
| SWU19534 | G_Chr10 | 51.555 | P_06 | 42289012 | 42289165 |
| SWU19460 | G_Chr10 | 51.61 | P_06 | 31815800 | 31815963 |
| SWU19489 | G_Chr10 | 51.61 | P_06 | 36007675 | 36007850 |
| SWU19514 | G_Chr10 | 51.618 | P_06 | 39882134 | 39882293 |
| SWU19523 | G_Chr10 | 51.622 | P_06 | 41150173 | 41150329 |
| SWU19400 | G_Chr10 | 51.76 | P_06 | 25600274 | 25600450 |
| SWU19397 | G_Chr10 | 51.78 | P_06 | 25289456 | 25289616 |
| SWU19572 | G_Chr10 | 51.929 | P_06 | 45702064 | 45702207 |
| SWU19548 | G_Chr10 | 52.102 | P_06 | 43306609 | 43306794 |
| SWU19685 | G_Chr10 | 55.196 | P_06 | 56541012 | 56541107 |
| SWU19683 | G_Chr10 | 55.437 | P_06 | 56403249 | 56403380 |
| SWU19684 | G_Chr10 | 55.815 | P_06 | 56540574 | 56540724 |
| SWU19710 | G_Chr10 | 57.028 | P_06 | 57652525 | 57652700 |
| SWU19717 | G_Chr10 | 60.821 | P_06 | 57922650 | 57922788 |
| SWU19725 | G_Chr10 | 60.824 | P_06 | 58200785 | 58200968 |
| SWU19718 | G_Chr10 | 61.089 | P_06 | 57923284 | 57923463 |
| SWU19772 | G_Chr10 | 78.281 | P_06 | 61272503 | 61272653 |
| SWU19772 | G_Chr10 | 78.281 | P_06 | 61245841 | 61246013 |
| SWU19812 | G_Chr10 | 87.052 | P_06 | 62919419 | 62919619 |
| SWU19814 | G_Chr10 | 87.852 | P_06 | 62945266 | 62945482 |
| SWU19816 | G_Chr10 | 88.409 | P_06 | 63044674 | 63044814 |
| SWU19848 | G_Chr10 | 97.068 | P_06 | 63500155 | 63500342 |
| SWU19836 | G_Chr10 | 103.563 | P_06 | 63904514 | 63904635 |
| SWU22383 | G_Chr13 | 45.492 | P_06 | 54569324 | 54569494 |
| SWU10025 | G_Chr01 | 0 | P_07 | 784936 | 785114 |
| SWU10022 | G_Chr01 | 4.62 | P_07 | 705214 | 705408 |
| SWU10014 | G_Chr01 | 6.038 | P_07 | 576866 | 577053 |
| SWU10064 | G_Chr01 | 21.737 | P_07 | 1780284 | 1780486 |
| SWU10076 | G_Chr01 | 26.228 | P_07 | 2415002 | 2415181 |
| SWU10076 | G_Chr01 | 26.228 | P_07 | 2395778 | 2395975 |
| SWU10237 | G_Chr01 | 39.038 | P_07 | 9776747 | 9776910 |
| SWU10135 | G_Chr01 | 40.968 | P_07 | 5558371 | 5558496 |
| SWU10135 | G_Chr01 | 40.968 | P_07 | 5309885 | 5310103 |
| SWU10141 | G_Chr01 | 41.402 | P_07 | 5484456 | 5484632 |
| SWU10144 | G_Chr01 | 42.133 | P_07 | 5591245 | 5591415 |
| SWU10166 | G_Chr01 | 43.883 | P_07 | 6694903 | 6695073 |
| SWU10181 | G_Chr01 | 46.475 | P_07 | 7055145 | 7055281 |
| SWU10177 | G_Chr01 | 46.58 | P_07 | 6953295 | 6953483 |
| SWU10219 | G_Chr01 | 48.48 | P_07 | 9271964 | 9272132 |
| SWU10235 | G_Chr01 | 49.387 | P_07 | 9615654 | 9615843 |
| SWU10320 | G_Chr01 | 51.388 | P_07 | 15439760 | 15439944 |
| SWU10315 | G_Chr01 | 51.439 | P_07 | 15229872 | 15230067 |
| SWU10321 | G_Chr01 | 52.475 | P_07 | 15445080 | 15445273 |
| SWU10441 | G_Chr01 | 57.098 | P_07 | 22657999 | 22658156 |
| SWU10464 | G_Chr01 | 58.653 | P_07 | 24786011 | 24786159 |
| SWU10458 | G_Chr01 | 58.671 | P_07 | 24438492 | 24438595 |
| SWU10498 | G_Chr01 | 59.071 | P_07 | 26983815 | 26983996 |
| SWU10488 | G_Chr01 | 59.157 | P_07 | 25822811 | 25822968 |
| SWU10481 | G_Chr01 | 59.335 | P_07 | 25597927 | 25598118 |
| SWU10492 | G_Chr01 | 59.359 | P_07 | 26207427 | 26207530 |
| SWU10470 | G_Chr01 | 59.392 | P_07 | 25033545 | 25033740 |
| SWU10590 | G_Chr01 | 60.949 | P_07 | 36714132 | 36714278 |
| SWU10569 | G_Chr01 | 61.079 | P_07 | 34774739 | 34774892 |
| SWU10640 | G_Chr01 | 61.409 | P_07 | 42725310 | 42725493 |
| SWU10625 | G_Chr01 | 61.623 | P_07 | 42090390 | 42090555 |
| SWU10622 | G_Chr01 | 61.796 | P_07 | 41758095 | 41758285 |
| SWU10652 | G_Chr01 | 62.234 | P_07 | 43578033 | 43578125 |
| SWU10657 | G_Chr01 | 62.563 | P_07 | 44939188 | 44939339 |
| SWU10657 | G_Chr01 | 62.563 | P_07 | 44663712 | 44663866 |
| SWU10680 | G_Chr01 | 63.591 | P_07 | 46093913 | 46094044 |
| SWU10664 | G_Chr01 | 64.043 | P_07 | 45354427 | 45354616 |
| SWU10697 | G_Chr01 | 64.695 | P_07 | 47409381 | 47409518 |
| SWU10690 | G_Chr01 | 65.244 | P_07 | 47010232 | 47010404 |
| SWU10756 | G_Chr01 | 70.554 | P_07 | 50348123 | 50348298 |
| SWU10764 | G_Chr01 | 73.399 | P_07 | 50600010 | 50600102 |
| SWU10484 | G_Chr01 | 74.831 | P_07 | 25771732 | 25771902 |
| SWU10761 | G_Chr01 | 76.269 | P_07 | 50560492 | 50560679 |
| SWU10849 | G_Chr01 | 88.137 | P_07 | 53297671 | 53297819 |
| SWU10867 | G_Chr01 | 95.357 | P_07 | 54003398 | 54003590 |
| SWU10889 | G_Chr01 | 100.883 | P_07 | 54870342 | 54870543 |
| SWU10894 | G_Chr01 | 102.761 | P_07 | 55146449 | 55146626 |
| SWU13644 | G_Chr04 | 7.975 | P_07 | 36653535 | 36653664 |
| SWU12734 | G_Chr02 | 15.036 | P_08 | 58884344 | 58884516 |
| SWU13240 | G_Chr04 | 0 | P_08 | 14691564 | 14691724 |
| SWU13829 | G_Chr04 | 0 | P_08 | 63954747 | 63954918 |
| SWU13830 | G_Chr04 | 0.454 | P_08 | 63958603 | 63958742 |
| SWU13798 | G_Chr04 | 2.652 | P_08 | 62584994 | 62585154 |
| SWU13805 | G_Chr04 | 3.559 | P_08 | 62867126 | 62867309 |
| SWU13816 | G_Chr04 | 4.66 | P_08 | 63210093 | 63210285 |
| SWU13787 | G_Chr04 | 6.233 | P_08 | 61895030 | 61895213 |
| SWU13781 | G_Chr04 | 6.479 | P_08 | 61692838 | 61692966 |
| SWU13779 | G_Chr04 | 6.567 | P_08 | 61597451 | 61597610 |
| SWU13760 | G_Chr04 | 7.97 | P_08 | 60783764 | 60783934 |
| SWU13644 | G_Chr04 | 7.975 | P_08 | 53962319 | 53962448 |
| SWU13720 | G_Chr04 | 11.871 | P_08 | 59105068 | 59105185 |
| SWU13670 | G_Chr04 | 14.577 | P_08 | 56213081 | 56213244 |
| SWU13671 | G_Chr04 | 15.871 | P_08 | 56251610 | 56251782 |
| SWU13672 | G_Chr04 | 15.915 | P_08 | 56252184 | 56252294 |
| SWU13667 | G_Chr04 | 16.349 | P_08 | 55893356 | 55893486 |
| SWU13631 | G_Chr04 | 17.185 | P_08 | 53527117 | 53527234 |
| SWU13622 | G_Chr04 | 17.659 | P_08 | 53181037 | 53181177 |
| SWU13620 | G_Chr04 | 17.93 | P_08 | 53117958 | 53118158 |
| SWU13597 | G_Chr04 | 18.787 | P_08 | 52036491 | 52036641 |
| SWU13602 | G_Chr04 | 18.787 | P_08 | 52168104 | 52168286 |
| SWU13592 | G_Chr04 | 19.984 | P_08 | 51838235 | 51838366 |
| SWU13610 | G_Chr04 | 20.197 | P_08 | 52600859 | 52601054 |
| SWU13544 | G_Chr04 | 20.895 | P_08 | 49140979 | 49141167 |
| SWU13544 | G_Chr04 | 20.895 | P_08 | 49139251 | 49139449 |
| SWU13545 | G_Chr04 | 21.241 | P_08 | 49192092 | 49192241 |
| SWU13554 | G_Chr04 | 21.401 | P_08 | 49628785 | 49628883 |
| SWU13513 | G_Chr04 | 22.646 | P_08 | 46774282 | 46774461 |
| SWU13520 | G_Chr04 | 22.646 | P_08 | 47179376 | 47179493 |
| SWU13523 | G_Chr04 | 22.647 | P_08 | 47293446 | 47293659 |
| SWU13412 | G_Chr04 | 24.463 | P_08 | 39490733 | 39490996 |
| SWU13412 | G_Chr04 | 24.463 | P_08 | 39490733 | 39490843 |
| SWU13398 | G_Chr04 | 24.853 | P_08 | 38261533 | 38261711 |
| SWU13375 | G_Chr04 | 25.148 | P_08 | 35839870 | 35840047 |
| SWU13293 | G_Chr04 | 25.375 | P_08 | 22729847 | 22729954 |
| SWU13292 | G_Chr04 | 25.38 | P_08 | 22674285 | 22674418 |
| SWU13296 | G_Chr04 | 25.38 | P_08 | 23000721 | 23000904 |
| SWU13334 | G_Chr04 | 25.38 | P_08 | 29756241 | 29756439 |
| SWU13297 | G_Chr04 | 25.392 | P_08 | 23117398 | 23117584 |
| SWU13370 | G_Chr04 | 25.458 | P_08 | 34957240 | 34957400 |
| SWU13266 | G_Chr04 | 25.548 | P_08 | 19127745 | 19127908 |
| SWU13369 | G_Chr04 | 25.595 | P_08 | 34934745 | 34934929 |
| SWU13274 | G_Chr04 | 25.701 | P_08 | 19688040 | 19688161 |
| SWU13228 | G_Chr04 | 26.067 | P_08 | 12919350 | 12919498 |
| SWU13251 | G_Chr04 | 26.661 | P_08 | 15863761 | 15863910 |
| SWU13217 | G_Chr04 | 27.051 | P_08 | 12175604 | 12175792 |
| SWU13264 | G_Chr04 | 27.618 | P_08 | 18938343 | 18938468 |
| SWU13165 | G_Chr04 | 28.388 | P_08 | 7626274 | 7626422 |
| SWU13133 | G_Chr04 | 29.121 | P_08 | 6844619 | 6844788 |
| SWU13130 | G_Chr04 | 29.532 | P_08 | 6606299 | 6606474 |
| SWU13129 | G_Chr04 | 29.618 | P_08 | 6604334 | 6604458 |
| SWU13117 | G_Chr04 | 31.124 | P_08 | 6112734 | 6112923 |
| SWU13082 | G_Chr04 | 37.949 | P_08 | 4228161 | 4228369 |
| SWU13048 | G_Chr04 | 41.072 | P_08 | 3109257 | 3109422 |
| SWU13013 | G_Chr04 | 58.203 | P_08 | 1865598 | 1865783 |
| SWU13008 | G_Chr04 | 59.229 | P_08 | 1791994 | 1792146 |
| SWU15003 | G_Chr06 | 9.061 | P_08 | 53826884 | 53826961 |
| SWU13865 | G_Chr09 | 0 | P_08 | 64806977 | 64807164 |
| SWU13887 | G_Chr09 | 12.515 | P_08 | 65751230 | 65751370 |
| SWU20590 | G_Chr11 | 41.34 | P_08 | 40738722 | 40738916 |
| SWU15054 | G_Chr01 | 61.469 | P_09 | 22446873 | 22447046 |
| SWU15145 | G_Chr03 | 2.45 | P_09 | 30569728 | 30569905 |
| SWU15145 | G_Chr03 | 2.45 | P_09 | 30563611 | 30563782 |
| SWU13644 | G_Chr04 | 7.975 | P_09 | 678922 | 679051 |
| SWU13990 | G_Chr05 | 23.206 | P_09 | 48512565 | 48512761 |
| SWU14987 | G_Chr06 | 0 | P_09 | 17299516 | 17299692 |
| SWU15145 | G_Chr06 | 9.117 | P_09 | 30569728 | 30569905 |
| SWU15145 | G_Chr06 | 0.56 | P_09 | 30563611 | 30563782 |
| SWU14858 | G_Chr06 | 2.587 | P_09 | 6183883 | 6184042 |
| SWU14854 | G_Chr06 | 3.271 | P_09 | 5840453 | 5840651 |
| SWU15054 | G_Chr06 | 5.936 | P_09 | 22446873 | 22447046 |
| SWU14909 | G_Chr06 | 6.338 | P_09 | 8969123 | 8969244 |
| SWU14939 | G_Chr06 | 8.234 | P_09 | 11758635 | 11758824 |
| SWU15003 | G_Chr06 | 9.061 | P_09 | 49959277 | 49959348 |
| SWU14987 | G_Chr06 | 0 | P_09 | 17299516 | 17299692 |
| SWU15057 | G_Chr06 | 9.183 | P_09 | 22296449 | 22296627 |
| SWU15006 | G_Chr06 | 9.224 | P_09 | 25817278 | 25817392 |
| SWU14996 | G_Chr06 | 9.232 | P_09 | 18160045 | 18160216 |
| SWU15076 | G_Chr06 | 9.242 | P_09 | 20180143 | 20180335 |
| SWU14978 | G_Chr06 | 9.329 | P_09 | 16012139 | 16012328 |
| SWU14989 | G_Chr06 | 9.426 | P_09 | 17357923 | 17358076 |
| SWU15037 | G_Chr06 | 9.644 | P_09 | 23953227 | 23953416 |
| SWU14940 | G_Chr06 | 9.739 | P_09 | 11837975 | 11838155 |
| SWU15130 | G_Chr06 | 11.355 | P_09 | 29797587 | 29797729 |
| SWU14853 | G_Chr06 | 11.469 | P_09 | 5836562 | 5836739 |
| SWU14848 | G_Chr06 | 11.793 | P_09 | 5747209 | 5747346 |
| SWU15146 | G_Chr06 | 12.911 | P_09 | 30602880 | 30603053 |
| SWU15156 | G_Chr06 | 13.278 | P_09 | 30971097 | 30971274 |
| SWU15156 | G_Chr06 | 13.278 | P_09 | 30978977 | 30979175 |
| SWU15167 | G_Chr06 | 14.759 | P_09 | 31448746 | 31448919 |
| SWU15215 | G_Chr06 | 23.014 | P_09 | 34004918 | 34005116 |
| SWU15252 | G_Chr06 | 28.646 | P_09 | 36153855 | 36154037 |
| SWU15265 | G_Chr06 | 29.01 | P_09 | 36611323 | 36611465 |
| SWU15302 | G_Chr06 | 31.733 | P_09 | 37736316 | 37736484 |
| SWU15335 | G_Chr06 | 33.652 | P_09 | 38953295 | 38953458 |
| SWU15382 | G_Chr06 | 37.055 | P_09 | 40837233 | 40837417 |
| SWU15396 | G_Chr06 | 37.456 | P_09 | 41346781 | 41346981 |
| SWU15395 | G_Chr06 | 37.488 | P_09 | 41329642 | 41329825 |
| SWU15384 | G_Chr06 | 37.569 | P_09 | 40917929 | 40918127 |
| SWU15383 | G_Chr06 | 37.851 | P_09 | 40868918 | 40869026 |
| SWU15399 | G_Chr06 | 38.544 | P_09 | 41572638 | 41572760 |
| SWU15403 | G_Chr06 | 39.416 | P_09 | 41750977 | 41751156 |
| SWU15409 | G_Chr06 | 40.644 | P_09 | 41994906 | 41995080 |
| SWU15406 | G_Chr06 | 40.646 | P_09 | 41961892 | 41962059 |
| SWU15416 | G_Chr06 | 41.615 | P_09 | 42235856 | 42236048 |
| SWU15440 | G_Chr06 | 43.092 | P_09 | 43049649 | 43049813 |
| SWU15438 | G_Chr06 | 43.595 | P_09 | 42893374 | 42893493 |
| SWU15453 | G_Chr06 | 45.742 | P_09 | 43533952 | 43534141 |
| SWU15459 | G_Chr06 | 46.269 | P_09 | 43923651 | 43923840 |
| SWU15469 | G_Chr06 | 47.407 | P_09 | 44208918 | 44209020 |
| SWU15477 | G_Chr06 | 48.936 | P_09 | 44614174 | 44614349 |
| SWU15485 | G_Chr06 | 49.855 | P_09 | 45222323 | 45222514 |
| SWU15484 | G_Chr06 | 50.289 | P_09 | 45208079 | 45208243 |
| SWU15496 | G_Chr06 | 54.984 | P_09 | 45759696 | 45759872 |
| SWU15503 | G_Chr06 | 56.511 | P_09 | 45994568 | 45994759 |
| SWU15503 | G_Chr06 | 56.511 | P_09 | 45977217 | 45977417 |
| SWU15505 | G_Chr06 | 56.922 | P_09 | 46015696 | 46015883 |
| SWU15508 | G_Chr06 | 57.515 | P_09 | 46075649 | 46075816 |
| SWU15520 | G_Chr06 | 59.414 | P_09 | 46381810 | 46381967 |
| SWU15536 | G_Chr06 | 61.464 | P_09 | 46976451 | 46976606 |
| SWU15538 | G_Chr06 | 62.278 | P_09 | 47046162 | 47046296 |
| SWU15548 | G_Chr06 | 63.652 | P_09 | 47420760 | 47420912 |
| SWU15544 | G_Chr06 | 64.213 | P_09 | 47332289 | 47332441 |
| SWU15193 | G_Chr07 | 28.58 | P_09 | 32631322 | 32631492 |
| SWU19087 | G_Chr10 | 38.212 | P_09 | 2598701 | 2598890 |
| SWU21354 | G_Chr12 | 50.57 | P_09 | 42887249 | 42887365 |
| SWU22383 | G_Chr13 | 45.492 | P_09 | 24971859 | 24972029 |
| SWU10135 | G_Chr01 | 40.968 | P_10 | 41741858 | 41742013 |
| SWU10135 | G_Chr01 | 40.968 | P_10 | 22950790 | 22950945 |
| SWU20737 | G_Chr11 | 0 | P_10 | 63353421 | 63353599 |
| SWU20727 | G_Chr11 | 2.898 | P_10 | 62980596 | 62980745 |
| SWU20725 | G_Chr11 | 3.43 | P_10 | 62910774 | 62910916 |
| SWU19858 | G_Chr11 | 3.636 | P_10 | 310484 | 310663 |
| SWU19852 | G_Chr11 | 5.79 | P_10 | 31297 | 31433 |
| SWU19861 | G_Chr11 | 7.335 | P_10 | 505257 | 505441 |
| SWU19864 | G_Chr11 | 8.56 | P_10 | 553425 | 553602 |
| SWU20713 | G_Chr11 | 12.48 | P_10 | 62351572 | 62351763 |
| SWU20709 | G_Chr11 | 13.023 | P_10 | 61897292 | 61897469 |
| SWU19875 | G_Chr11 | 14.112 | P_10 | 983682 | 983779 |
| SWU20688 | G_Chr11 | 20.4 | P_10 | 61247543 | 61247719 |
| SWU19903 | G_Chr11 | 28.504 | P_10 | 1903140 | 1903325 |
| SWU19902 | G_Chr11 | 29.039 | P_10 | 1890549 | 1890685 |
| SWU20639 | G_Chr11 | 34.04 | P_10 | 58456403 | 58456508 |
| SWU19923 | G_Chr11 | 35.939 | P_10 | 2671902 | 2672061 |
| SWU20590 | G_Chr11 | 41.34 | P_10 | 56338273 | 56338461 |
| SWU20588 | G_Chr11 | 41.503 | P_10 | 56247186 | 56247319 |
| SWU20591 | G_Chr11 | 41.805 | P_10 | 56356645 | 56356815 |
| SWU20572 | G_Chr11 | 43.777 | P_10 | 55547862 | 55548044 |
| SWU20576 | G_Chr11 | 43.958 | P_10 | 55564632 | 55564802 |
| SWU20577 | G_Chr11 | 44.122 | P_10 | 55658916 | 55659068 |
| SWU19937 | G_Chr11 | 45.346 | P_10 | 3569594 | 3569784 |
| SWU19940 | G_Chr11 | 45.59 | P_10 | 3637865 | 3637963 |
| SWU20517 | G_Chr11 | 48.304 | P_10 | 51768739 | 51768921 |
| SWU20528 | G_Chr11 | 48.794 | P_10 | 52308673 | 52308809 |
| SWU20449 | G_Chr11 | 50.739 | P_10 | 47008628 | 47008819 |
| SWU20444 | G_Chr11 | 50.878 | P_10 | 46716852 | 46717045 |
| SWU20407 | G_Chr11 | 52.407 | P_10 | 42152892 | 42153048 |
| SWU20430 | G_Chr11 | 53.119 | P_10 | 45219260 | 45219407 |
| SWU20214 | G_Chr11 | 53.813 | P_10 | 20711078 | 20711265 |
| SWU20240 | G_Chr11 | 53.828 | P_10 | 23028006 | 23028164 |
| SWU20240 | G_Chr11 | 53.828 | P_10 | 23026633 | 23026791 |
| SWU20360 | G_Chr11 | 53.828 | P_10 | 35552015 | 35552191 |
| SWU20222 | G_Chr11 | 53.839 | P_10 | 21211291 | 21211447 |
| SWU20225 | G_Chr11 | 53.855 | P_10 | 21358076 | 21358261 |
| SWU20283 | G_Chr11 | 53.855 | P_10 | 27189259 | 27189421 |
| SWU20303 | G_Chr11 | 53.855 | P_10 | 29958761 | 29958909 |
| SWU20375 | G_Chr11 | 53.855 | P_10 | 37369278 | 37369457 |
| SWU20375 | G_Chr11 | 53.855 | P_10 | 37369239 | 37369457 |
| SWU20394 | G_Chr11 | 53.855 | P_10 | 41415143 | 41415256 |
| SWU20426 | G_Chr11 | 53.855 | P_10 | 44491591 | 44491721 |
| SWU20235 | G_Chr11 | 54.048 | P_10 | 22765449 | 22765597 |
| SWU20206 | G_Chr11 | 55.01 | P_10 | 19943315 | 19943487 |
| SWU20155 | G_Chr11 | 55.943 | P_10 | 15790460 | 15790662 |
| SWU20137 | G_Chr11 | 56.095 | P_10 | 14465650 | 14465743 |
| SWU20171 | G_Chr11 | 56.099 | P_10 | 17139026 | 17139183 |
| SWU20158 | G_Chr11 | 56.105 | P_10 | 16107474 | 16107641 |
| SWU20161 | G_Chr11 | 56.131 | P_10 | 16450186 | 16450361 |
| SWU20143 | G_Chr11 | 56.259 | P_10 | 14707963 | 14708136 |
| SWU20099 | G_Chr11 | 56.866 | P_10 | 12305252 | 12305458 |
| SWU20117 | G_Chr11 | 57.673 | P_10 | 13001360 | 13001454 |
| SWU20080 | G_Chr11 | 58.823 | P_10 | 10962437 | 10962653 |
| SWU20073 | G_Chr11 | 58.998 | P_10 | 10227495 | 10227673 |
| SWU20054 | G_Chr11 | 59.318 | P_10 | 9381804 | 9381964 |
| SWU20017 | G_Chr11 | 59.34 | P_10 | 7634710 | 7634867 |
| SWU20062 | G_Chr11 | 60.089 | P_10 | 9739138 | 9739306 |
| SWU19986 | G_Chr11 | 60.665 | P_10 | 5664487 | 5664616 |
| SWU20060 | G_Chr11 | 61.981 | P_10 | 9731368 | 9731540 |
| SWU19964 | G_Chr11 | 63.273 | P_10 | 4771929 | 4772054 |
| SWU19957 | G_Chr11 | 64.604 | P_10 | 4439554 | 4439714 |
| SWU10135 | G_Chr01 | 40.968 | P_11 | 2863348 | 2863472 |
| SWU13644 | G_Chr04 | 7.975 | P_11 | 11774697 | 11774822 |
| SWU14540 | G_Chr05 | 42.165 | P_11 | 55230363 | 55230536 |
| SWU15811 | G_Chr07 | 0 | P_11 | 7765833 | 7766021 |
| SWU15834 | G_Chr07 | 4.34 | P_11 | 8913649 | 8913733 |
| SWU15841 | G_Chr07 | 5.923 | P_11 | 9247740 | 9247927 |
| SWU15841 | G_Chr07 | 5.923 | P_11 | 9247740 | 9247927 |
| SWU15851 | G_Chr07 | 6.206 | P_11 | 9534636 | 9534763 |
| SWU15858 | G_Chr07 | 6.693 | P_11 | 10217888 | 10218008 |
| SWU15873 | G_Chr07 | 7.187 | P_11 | 10928325 | 10928520 |
| SWU15879 | G_Chr07 | 8.099 | P_11 | 11081446 | 11081609 |
| SWU15883 | G_Chr07 | 8.629 | P_11 | 11211524 | 11211674 |
| SWU15881 | G_Chr07 | 8.763 | P_11 | 11145073 | 11145179 |
| SWU15880 | G_Chr07 | 8.969 | P_11 | 11113715 | 11113927 |
| SWU15890 | G_Chr07 | 9.906 | P_11 | 11510469 | 11510622 |
| SWU15892 | G_Chr07 | 10.021 | P_11 | 11627398 | 11627580 |
| SWU15900 | G_Chr07 | 10.26 | P_11 | 11980585 | 11980775 |
| SWU15944 | G_Chr07 | 14.58 | P_11 | 13756550 | 13756723 |
| SWU15954 | G_Chr07 | 17.757 | P_11 | 13883409 | 13883549 |
| SWU16069 | G_Chr07 | 27.244 | P_11 | 20606352 | 20606538 |
| SWU16066 | G_Chr07 | 27.443 | P_11 | 20432002 | 20432161 |
| SWU16059 | G_Chr07 | 27.484 | P_11 | 19984095 | 19984286 |
| SWU16057 | G_Chr07 | 27.499 | P_11 | 19912013 | 19912143 |
| SWU16104 | G_Chr07 | 29.46 | P_11 | 23751790 | 23751955 |
| SWU16143 | G_Chr07 | 30.944 | P_11 | 26119121 | 26119238 |
| SWU16148 | G_Chr07 | 31.43 | P_11 | 26593022 | 26593203 |
| SWU16178 | G_Chr07 | 32.657 | P_11 | 29191858 | 29192010 |
| SWU16185 | G_Chr07 | 32.918 | P_11 | 29536862 | 29537031 |
| SWU14289 | G_Chr07 | 33.571 | P_11 | 28450135 | 28450325 |
| SWU16199 | G_Chr07 | 33.785 | P_11 | 31270651 | 31270743 |
| SWU16259 | G_Chr07 | 34.546 | P_11 | 39219311 | 39219463 |
| SWU16295 | G_Chr07 | 34.723 | P_11 | 46257784 | 46257979 |
| SWU16269 | G_Chr07 | 34.848 | P_11 | 43865887 | 43866041 |
| SWU16204 | G_Chr07 | 35.155 | P_11 | 31554915 | 31555069 |
| SWU16141 | G_Chr07 | 35.438 | P_11 | 26058192 | 26058326 |
| SWU16286 | G_Chr07 | 36.187 | P_11 | 45207329 | 45207500 |
| SWU16286 | G_Chr07 | 36.187 | P_11 | 45205597 | 45205768 |
| SWU16343 | G_Chr07 | 36.568 | P_11 | 49935276 | 49935465 |
| SWU16330 | G_Chr07 | 37.317 | P_11 | 49197838 | 49197971 |
| SWU16531 | G_Chr07 | 54.701 | P_11 | 60739460 | 60739580 |
| SWU16535 | G_Chr07 | 56.362 | P_11 | 59769311 | 59769456 |
| SWU16534 | G_Chr07 | 56.751 | P_11 | 59777093 | 59777266 |
| SWU16553 | G_Chr07 | 60.492 | P_11 | 61808983 | 61809171 |
| SWU16555 | G_Chr07 | 60.937 | P_11 | 61834929 | 61835109 |
| SWU16562 | G_Chr07 | 61.247 | P_11 | 62405729 | 62405904 |
| SWU16566 | G_Chr07 | 62.013 | P_11 | 62571893 | 62572046 |
| SWU16586 | G_Chr07 | 69.003 | P_11 | 63208903 | 63209148 |
| SWU16586 | G_Chr07 | 69.003 | P_11 | 63203110 | 63203334 |
| SWU16992 | G_Chr08 | 66.209 | P_11 | 31854112 | 31854210 |
| SWU16920 | G_Chr08 | 66.803 | P_11 | 54937414 | 54937598 |
| SWU16920 | G_Chr08 | 66.803 | P_11 | 40219785 | 40219977 |
| SWU19087 | G_Chr10 | 38.212 | P_11 | 44109884 | 44110072 |
| SWU16204 | G_Chr10 | 73.246 | P_11 | 31554915 | 31555069 |
| SWU22383 | G_Chr13 | 45.492 | P_11 | 52513877 | 52514046 |
| SWU10135 | G_Chr01 | 40.968 | P_12 | 42070234 | 42070387 |
| SWU10135 | G_Chr01 | 40.968 | P_12 | 42069423 | 42069585 |
| SWU10235 | G_Chr01 | 49.387 | P_12 | 36111199 | 36111397 |
| SWU14306 | G_Chr03 | 32.028 | P_12 | 38546864 | 38546980 |
| SWU13644 | G_Chr04 | 7.975 | P_12 | 49960959 | 49961086 |
| SWU14306 | G_Chr05 | 27.127 | P_12 | 38546864 | 38546980 |
| SWU17618 | G_Chr08 | 0 | P_12 | 58644627 | 58644813 |
| SWU17619 | G_Chr08 | 2.874 | P_12 | 58716504 | 58716676 |
| SWU17620 | G_Chr08 | 4.06 | P_12 | 58725742 | 58725869 |
| SWU17610 | G_Chr08 | 6.42 | P_12 | 58266371 | 58266588 |
| SWU17599 | G_Chr08 | 11.098 | P_12 | 58021409 | 58021598 |
| SWU17602 | G_Chr08 | 11.247 | P_12 | 58108857 | 58109032 |
| SWU17598 | G_Chr08 | 11.5 | P_12 | 57999781 | 57999970 |
| SWU17576 | G_Chr08 | 17.114 | P_12 | 57471861 | 57471988 |
| SWU17562 | G_Chr08 | 20.189 | P_12 | 57219098 | 57219212 |
| SWU17525 | G_Chr08 | 35.006 | P_12 | 56050018 | 56050162 |
| SWU17512 | G_Chr08 | 37.429 | P_12 | 55512401 | 55512598 |
| SWU17494 | G_Chr08 | 38.242 | P_12 | 54764577 | 54764719 |
| SWU17485 | G_Chr08 | 40.487 | P_12 | 54152476 | 54152646 |
| SWU17483 | G_Chr08 | 40.731 | P_12 | 54106386 | 54106562 |
| SWU17442 | G_Chr08 | 44.67 | P_12 | 52938463 | 52938656 |
| SWU17360 | G_Chr08 | 51.511 | P_12 | 49207017 | 49207208 |
| SWU17340 | G_Chr08 | 53.354 | P_12 | 47957132 | 47957270 |
| SWU17128 | G_Chr08 | 54.989 | P_12 | 37655634 | 37655800 |
| SWU17278 | G_Chr08 | 57.248 | P_12 | 44987694 | 44987880 |
| SWU17206 | G_Chr08 | 58.952 | P_12 | 41526875 | 41527018 |
| SWU17202 | G_Chr08 | 61.085 | P_12 | 41383554 | 41383737 |
| SWU17186 | G_Chr08 | 61.699 | P_12 | 40377261 | 40377440 |
| SWU17179 | G_Chr08 | 62.35 | P_12 | 40175690 | 40175798 |
| SWU17155 | G_Chr08 | 63.228 | P_12 | 39219520 | 39219717 |
| SWU17131 | G_Chr08 | 63.671 | P_12 | 37754228 | 37754403 |
| SWU17127 | G_Chr08 | 63.927 | P_12 | 37643170 | 37643340 |
| SWU17089 | G_Chr08 | 65.919 | P_12 | 34806146 | 34806308 |
| SWU16960 | G_Chr08 | 66.25 | P_12 | 19346046 | 19346187 |
| SWU16965 | G_Chr08 | 66.252 | P_12 | 19647633 | 19647732 |
| SWU16899 | G_Chr08 | 66.435 | P_12 | 13985960 | 13986155 |
| SWU16888 | G_Chr08 | 66.444 | P_12 | 27385842 | 27386021 |
| SWU16870 | G_Chr08 | 66.447 | P_12 | 12233274 | 12233456 |
| SWU16920 | G_Chr08 | 66.803 | P_12 | 15672007 | 15672198 |
| SWU16916 | G_Chr08 | 67.516 | P_12 | 15248022 | 15248209 |
| SWU16856 | G_Chr08 | 68.071 | P_12 | 11130126 | 11130250 |
| SWU16746 | G_Chr08 | 70.75 | P_12 | 4070835 | 4071007 |
| SWU16794 | G_Chr08 | 70.761 | P_12 | 7055813 | 7055990 |
| SWU16795 | G_Chr08 | 71.118 | P_12 | 7350013 | 7350151 |
| SWU16881 | G_Chr08 | 72.406 | P_12 | 28191487 | 28191677 |
| SWU16845 | G_Chr08 | 76.304 | P_12 | 10700262 | 10700394 |
| SWU16729 | G_Chr08 | 80.053 | P_12 | 3208857 | 3208967 |
| SWU10135 | G_Chr01 | 40.968 | P_13 | 19540994 | 19541128 |
| SWU15145 | G_Chr03 | 2.45 | P_13 | 6551682 | 6551840 |
| SWU13644 | G_Chr04 | 7.975 | P_13 | 52356809 | 52356938 |
| SWU15145 | G_Chr06 | 0.56 | P_13 | 6551682 | 6551840 |
